# *Arabidopsis thaliana* AUCSIA-1 Regulates Auxin Biology and Physically Interacts with a Kinesin-Related Protein

**DOI:** 10.1371/journal.pone.0041327

**Published:** 2012-07-20

**Authors:** Barbara Molesini, Tiziana Pandolfini, Youry Pii, Arthur Korte, Angelo Spena

**Affiliations:** 1 Department of Biotechnology, University of Verona, Verona, Italy; 2 WissenschaftZentrum WeihenstephanTechnische Universitaet Muenchen, Freising, Germany; Iwate University, Japan

## Abstract

*Aucsia* is a green plant gene family encoding 44–54 amino acids long miniproteins. The sequenced genomes of most land plants contain two *Aucsia* genes. RNA interference of both tomato (*Solanum lycopersicum*) *Aucsia* genes (*SlAucsia*-1 and *SlAucsia*-2) altered auxin sensitivity, auxin transport and distribution; it caused parthenocarpic development of the fruit and other auxin-related morphological changes. Here we present data showing that the *Aucsia*-1 gene of *Arabidopsis thaliana* alters, by itself, root auxin biology and that the AtAUCSIA-1 miniprotein physically interacts with a kinesin-related protein. The *AtAucsia*-1 gene is ubiquitously expressed, although its expression is higher in roots and inflorescences in comparison to stems and leaves. Two allelic mutants for *AtAucsia*-1 gene did not display visible root morphological alterations; however both basipetal and acropetal indole-3-acetic acid (IAA) root transport was reduced as compared with wild-type plants. The transcript steady state levels of the auxin efflux transporters ATP BINDING CASSETTE subfamily B (ABCB) *ABCB1*, *ABCB4* and *ABCB19* were reduced in *ataucsia*-1 plants. In *ataucsia*-1 mutant, lateral root growth showed an altered response to i) exogenous auxin, ii) an inhibitor of polar auxin transport and iii) ethylene. Overexpression of *AtAucsia*-1 inhibited primary root growth. *In vitro* and *in vivo* protein-protein interaction experiments showed that AtAUCSIA-1 interacts with a 185 amino acids long fragment belonging to a 2712 amino acids long protein of unknown function (*At4g31570*). Bioinformatics analysis indicates that the AtAUCSIA-1 interacting protein (AtAUCSIA-1IP) clusters with a group of CENP-E kinesin-related proteins. Gene ontology predictions for the two proteins are consistent with the hypothesis that the AtAUCSIA-1/AtAUCSIA-1IP complex is involved in the regulation of the cytoskeleton dynamics underlying auxin biology.

## Introduction

The *Aucsia* gene family has been discovered and functionally identified in tomato (*Solanum lycopersicum*) by showing its role in both vegetative and reproductive development [1]. The two *Aucsia* genes of tomato, *SlAucsia*-1 and *SlAucsia*-2, are ubiquitously expressed and code for 53 amino acids long miniproteins. The analysis of *Aucsia*-silenced tomato plants demonstrated that *Aucsia* genes have a role in fruit initiation, leaf development, adventitious root formation and root growth [1]. *Aucsia*-silenced tomato plants showed a reduced polar auxin transport (PAT) in the roots and displayed modifications related to a modified content and/or distribution of auxin, such as an altered sensitivity to the PAT inhibitor N-1-napthylphthalamic acid (1-NPA), reduced rhizogenesis in response to auxin, parthenocarpic development of the fruit and morphological alterations of the leaves [1]. Thus, in tomato the two *Aucsia* genes are involved in the control of auxin biology.

Auxin is a phytohormone regulating many plant developmental and physiological processes in both the sporophyte [2] and gametophyte [3]. The predominant form of auxin is indole-3-acetic acid (IAA), a simple molecule that during green plant evolution became locally synthesized [4] and transported from cell to cell via polar transport [5]. IAA is the only phytohormone transported in a polar manner [6], [7]. IAA is also transported passively with the phloem sap, but it is PAT to be biologically relevant [5], [7].

Auxin biosynthesis takes place in all plant organs, is localized in few cells and regulated in response to environmental and developmental cues [4]. Besides localized auxin biosynthesis, the major determinant of differential auxin distribution is PAT, both at short and long distance [8]. For example, IAA synthesised in young expanding leaves at the shoot apex is transported to the root creating a morphogenetic gradient [7], [9], [10], [11], [12], [13]. PAT directionality is provided through transmembrane transporters acting as either efflux or influx carriers [6], [7], [14], [15]. Plants are endowed with different types of auxin transporters. In *Arabidopsis*, eight genes code for PIN-FORMED (PIN) proteins, but only five, i.e. PIN1-subfamily (long-PINs) are IAA efflux transporters localized at the plasma membrane, whilst the other three PINs are localized to the endoplasmic reticulum (ER) and are likely involved in IAA intracellular homeostasis [16]. Three ATP-BINDING CASSETTE subfamily B proteins (ABCB1, ABCB4, and ABCB19) are IAA transporters localized at the plasma membrane [17]. Two ATP-BINDING CASSETTE subfamily G transporters (ABCG36 and ABCG37) transport indole-3-butyric acid (IBA), a precursor of IAA, outside the cells [18]. Thus, auxin efflux and consequently auxin streams flow mainly via long-PIN [6] and ABCB IAA exporters [14], [19] acting both independently and synergistically at the plasma membrane [15], [19]. The uptake of undissociated IAA takes place via diffusion, however anionic IAA influx takes place via IAA symporters AUXIN RESISTANT 1/LIKE AUXIN RESISTANT 1–3 (AUX1/LAX1-3) and, under some conditions, via ABCB4 [17]. In the root, PIN1 and ABCB19 are the main players of acropetal auxin transport along the stele, while AUX1, PIN2 and ABCB4 mediate basipetal auxin transport from the root apex [17]. Lateral root formation depends on the auxin stream flowing toward the root [17].

Relevant to auxin biology in vascular plants it has been the discovery that long-PIN auxin exporters are often polar localized [20], [21] and that they can change localization in response to exogenous and endogenous cues taking place during development [22], in response to environmental changes [23] and in response to auxin [24], [25]. PIN transporters are considered to be first transported to the plasma membrane in a non-polar way and then after endocytosis and transcytosis to be polar localized [26], [27]. PIN proteins are rapidly and constitutively recycled between the plasma membrane and endosome compartments, whilst ABCB transporters behaves as less dynamic plasma membrane proteins [28]. Yet, ABCB19 interacts with PIN1 [15], [28]. ABCB1 and ABCB19 appear to function primarily in the maintenance of long-distance auxin streams and movement of auxin out of apical tissues [19], [29].

PAT control is complex. Among the mechanisms and molecular players, a relevant role in PAT control is played by a group of plant-specific kinases [30], [31]. For example, the kinase PINOID (PID) phosphorylates PIN1-type proteins conferring apical localization [32]. Another mechanism of controlling PAT is PIN ubiquitination and selective degradation [33]. Endocytosis and endomembrane trafficking are crucial for PAT and both involve the actin cytoskeleton [34]. In this regard, Robert and colleagues [35] have shown that the AUXIN BINDING PROTEIN 1 (ABP1) auxin receptor, present in the apoplast, is necessary for the appropriate assembly of the vesicle coat protein clathrin at the plasma membrane of root cells and consequently for endocytosis. Concomitantly, Xu and colleagues [36] have shown in pavement cells of the leaf that two different and antagonistic Rho Of Plants (ROP) signaling pathways, both depending on ABP1, determine actin-mediated lobe formation in one cell and tubulin-driven indentation in its adjacent cell. These findings indicate that auxin sensing in the apoplast via ABP1 is connected to endocytosis and to the regulation of cytoskeleton dynamics based on actin filaments and microtubules.

*Aucsia* homologous genes are widespread in the green plant lineage, from unicellular algae to angiosperms, and encode small proteins ranging from 44 to 54 amino acids. The genome of *Arabidopsis thaliana* contains two *Aucsia* genes. According to “The Arabidopsis Information Resource” (www.arabidopsis.org) *Aucsia* genes are ubiquitously expressed and are annotated as encoding components of the endomembrane system. This study extends the knowledge of the biological role of *Aucsia* family by dissecting the activity of *Aucsia*-1 gene in *A.thaliana*. The function of *AtAucsia*-1 has been first studied by using knockout T-DNA insertional mutants and *AtAucsia*-1 overexpressing lines, and then by the identification of an AtAUCSIA-1 interacting partner. Our data indicate that *AtAucsia*-1, by itself, is involved in root auxin biology and transport and that one interacting partner (AtAUCSIA-1IP) is a kinesin-related protein, putatively involved in cytoskeleton-based intracellular movements. The physical interaction of the two proteins highlighted by Yeast-Two-Hybrid (Y2H) assay has been confirmed *in vivo* by bimolecular fluorescence complementation (BiFC) and *in vitro* by pull-down binding experiments. Based on these data, a not yet explored connection between auxin biology and the cytoskeleton is revealed and a hypothetical role for AtAUCSIA-1 miniprotein in cytoskeleton dynamics is proposed.

## Results

### Identification and Genetic Characterisation of *ataucsia*-1 Mutants

**Figure 1:**
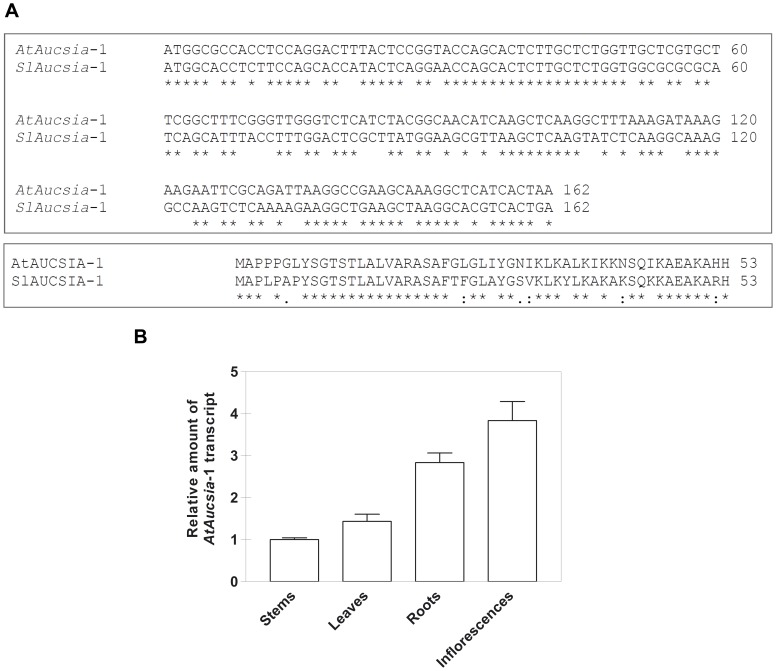
*Arabidopsis thaliana Aucsia*-1 coding sequence and expression in different organs. (A) Pairwise alignments of *Aucsia*-1 coding sequences from *A. thaliana and Solanum Lycopersicon* (upper) and comparison of the corresponding amino acid sequences (lower). * identical in all sequences, : for conserved substitutions; . for semi-conserved substitutions. (B) Expression pattern analysis of *AtAucsia*-1 in various tissues of wild-type adult plants assessed by quantitative real-time PCR (qRT-PCR). The expression levels were normalized using actin as endogenous control gene and the relative expression ratios were calculated using stems as calibrator sample. The values reported are means ± standard error (SE) (n = 3).

A search for tomato *Aucsia*-1 homologous gene in the *Arabidopsis thaliana* genome identified a candidate gene of unknown function (Figure 1A), named *AtAucsia*-1 (*At3g01130*), located in the chromosome 3, and annotated as component of the endomembrane system (http://www.arabidopsis.org/). *AtAucsia*-1 gene comprises 3 exons encoding a peptide of 53 amino acids (5546.5 Da). Quantitative RT-PCR (qRT-PCR), carried out on adult *A. thaliana* plants, showed that *AtAucsia*-1 gene is ubiquitously expressed, and yet relatively more abundant in inflorescences and roots (Figure 1B).

In *A. thaliana* a second *Aucsia* gene is present (*AtAucsia*-2; *At5g15320*). *AtAucsia*-2 coding region sequence is 81% identical to that of *AtAucsia*-1 (Figure S1). In tomato, the expression of a hairpin construct specifically designed to induce RNA interference (RNAi) of the *SlAucsia*-1 gene, also elicited the down regulation, albeit less strong (a 60% reduction in the expression level), of *SlAucsia*-2. This result is most likely due to the high sequence homology (i.e. 85%) between the two *SlAucsia* coding regions [1]. Therefore, to identify the biological role of *AtAucsia*-1, we have searched for null mutants (i.e. knock-out) instead of using an approach based on RNAi (i.e. knock-down).

**Figure 2:**
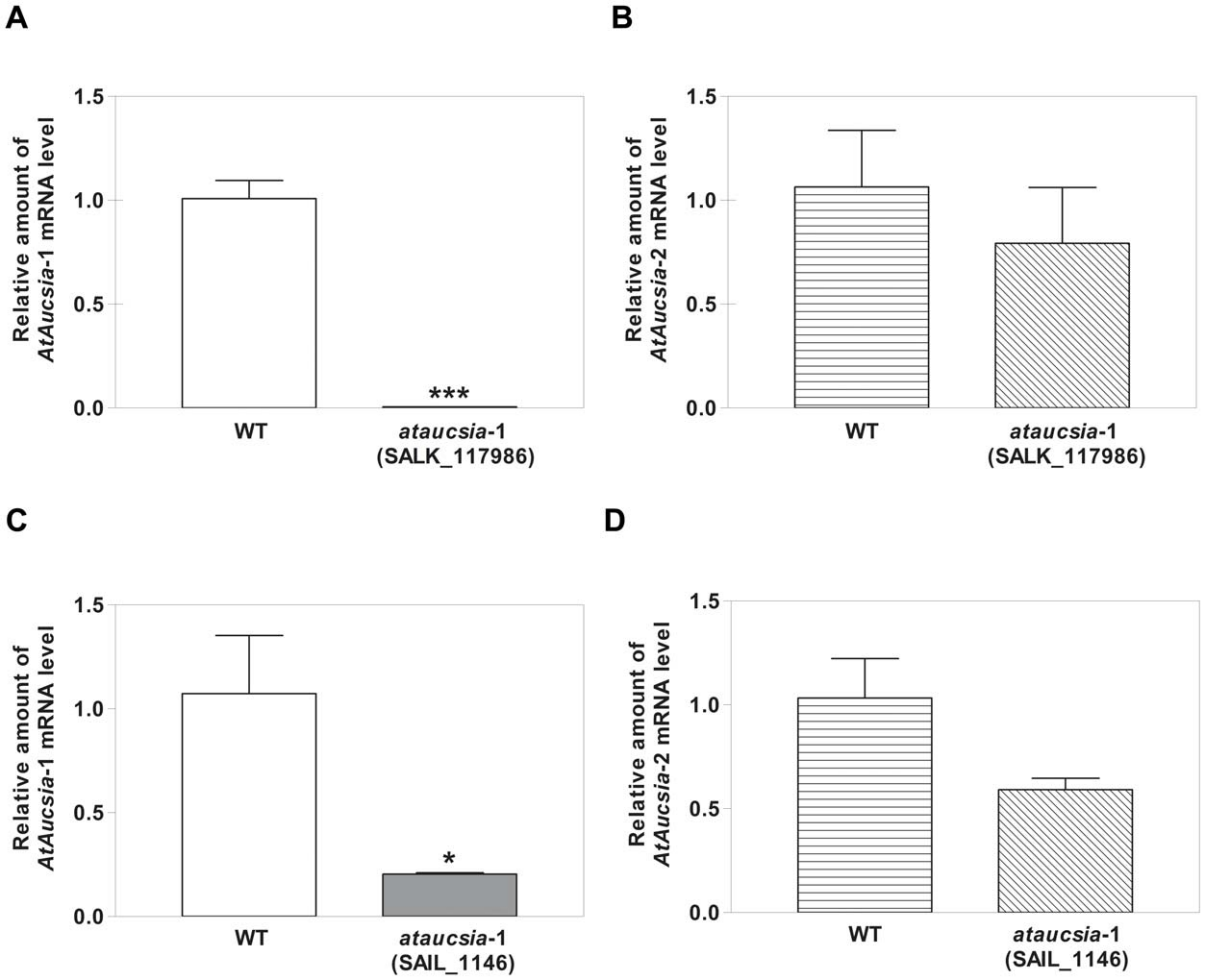
*Aucsia* gene expressions in *ataucsia*-1 mutants. (A) and (C) *AtAucsia*-1 mRNA level in *ataucsia*-1 mutants SALK_117986 and SAIL_1146, respectively. The relative mRNA level of *AtAucsia*-1 was assessed by qRT-PCR in comparison to the level of *AtAucsia*-1 in wild-type plants. (B) and (D) *AtAucsia*-2 mRNA level in *ataucsia*-1 mutants SALK_117986 and SAIL_1146, respectively. The relative mRNA levels of *AtAucsia*-2 was evaluated by qRT-PCR and compared with the level of *AtAucsia*-2 in wild-type plants. The values reported are means ± standard error (SE) (n = 3). Student's *t*-test was applied. *P<0.05; ***P<0.001 versus wild-type.

Two separate populations of T-DNA insertional mutants (SALK_117986 and SAIL_1146) were obtained from the SALK collection [37], and screened by PCR for the presence of T-DNA insertions in the *AtAucsia*-1 gene. The T-DNA insertion site in the SALK_117986 mutant line is located at the second exon (2 bp upstream the 3′-end of the exon) while for the second line (i.e. SAIL_1146), the T-DNA integration event occurs in the promoter sequence, 38 bases upstream the initiation of transcription. Homozygous lines for each allele were identified by genomic PCR. Quantitative RT-PCR (qRT-PCR) analyses were performed on these mutant lines in order to check *AtAucsia*-1 transcript level. The mRNA expression of *AtAucsia*-1 was completely abolished in *ataucsia*-1 mutant (SALK_117986) in comparison to wild-type plants and was 5 fold reduced (i.e. 19% of wild type steady state level) in the second mutant line (SAIL_1146) (Figure 2A and 2C, respectively). *AtAucsia*-2 mRNA steady state level was not significantly changed in both mutants (Figure 2B and 2D).

Compared with wild-type seedlings, homozygous mutant lines showed similar germination rate and no obvious morphological differences in vegetative growth and reproductive organ development as well as in seeds production.

### *ataucsia*-1 Mutants Exhibit Reduced Polar Auxin Transport

**Figure 3:**
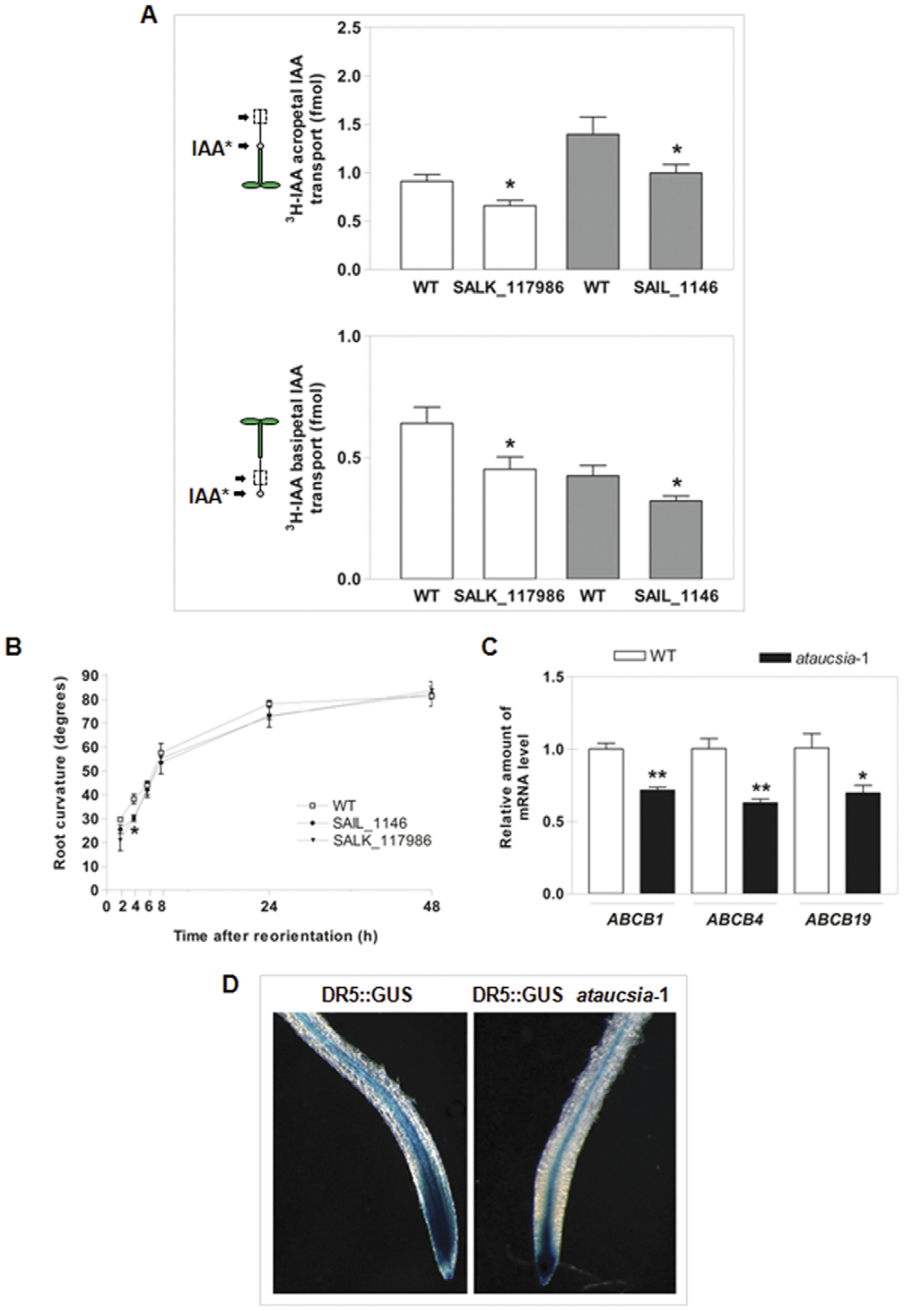
Polar auxin transport in *ataucsia*-1 mutants. (A) Auxin transport measurement in 5 days old wild-type and *ataucsia*-1 mutant seedlings. (Upper) For acropetal IAA transport assay, an agar drop containing ^3^H-IAA was applied just below the root–shoot junction. (Lower) For basipetal IAA transport assay, ^3^H-IAA agar drop was positioned to the apical 0.5 mm of the root tip. The schematic drawings on the left represent the mode of ^3^H-IAA application. The boxes indicate the segment in which radioactivity was measured. Results are reported as means ± SE (n = 3) 15–20 seedlings per replicate. (B) Time course analysis of gravitropic curvature in *ataucsia*-1 mutant and wild-type roots. The root bending was calculated at 2, 4, 6, 8, 24 and 48 h after 90° reorientation. Results are reported as means ± SE (n = 3) 15–20 seedlings per replicate. (C) Steady state levels of *ABCB1*, *ABCB4*, and *ABCB19* transcripts in wild-type and *ataucsia*-1 null mutant roots (SALK_117986) evaluated by qRT-PCR. The expression levels were normalized using actin as endogenous control. Relative transcript level for each ABCB gene is the ratio between the expression levels in *ataucsia*-1 mutant and wild-type roots. The values reported are means ± SE (n = 3). (D) Auxin activity in *ataucsia*-1×DR5::GUS roots. (Left) Wild-type roots expressing DR5::GUS. (Right) *ataucsia*-1 knock-out line expressing DR5::GUS. Data reported in panel A, B, and C were analyzed by Student's *t*-test. *P<0.05; **P<0.01; ***P<0.001 versus wild-type seedlings.

*Aucsia*-silenced tomato plants were impaired in root polar auxin transport (PAT) in comparison with wild-type control plants. Thus, to determine if *AtAucsia*-1 gene is functionally required in PAT, we assayed auxin polar flow in the two mutant lines and in wild-type seedlings. Acropetal and basipetal auxin transport experiments were performed by applying ^3^H-IAA at 100 nM concentration just below the root-shoot junction and at primary root tip, respectively. We quantified the level of labelled IAA moving from the site of application into a specific segment of the root (see Material and Methods). Approximately a 30% reduction of both basipetal and acropetal transport was observed in *ataucsia*-1 mutant roots (SALK_117986) as compared with wild-type roots (Figure 3A). Also in the second mutant (SAIL_1146) root PAT was perturbed. A 25% and 30% reduction in basipetal and acropetal transport, respectively, was detected (Figure 3A). Previous studies have reported that mutants with altered auxin transport often showed modified gravity responses [38]. The gravitropic response of the *ataucsia*-1 mutants was evaluated in 5 days-old seedlings. The kinetic of root gravitropic response was investigated over a 48-h period. As shown in figure 3B, the root tips of *ataucsia*-1 mutants and wild-type displayed a normal gravitropic bending at long times after reorientation (i.e 48 h). However, at shorter times (i.e. 4 h) after reorientation *ataucsia*-1 mutant roots showed a 26% reduction in curvature as compared with wild-type (Figure 3B).

The altered PAT prompted us to examine whether *AtAucsia*-1 mutation affects the gene expression of any auxin transporters. QRT-PCR analysis performed on null *ataucsia*-1 mutant (SALK_ 117986) revealed a 30–35% decrease in the steady state levels of *ABCB1*, *ABCB4* and *ABCB19* mRNAs in the root of *ataucsia*-1 as compared to wild-type (Figure 3C). No significant differences in transcript levels of *PIN1*, *PIN2*, *PIN3*, and *PIN7* were detected (data not shown). Auxin distribution and action could also be influenced by the activity of ABCG-type transporters (ABCG36 and ABCG37) that move out of the cells the IAA precursor indole-3-butyric acid (IBA) [18], [39]. The steady state levels of *ABCG36* and *ABCG37* transcripts were unchanged in *ataucsia*-1 mutant roots as compared with wild-type roots (data not shown) as well as the steady stale level of *AUX1* symporter transcript. To provide further indication that the *AtAucsia*-1 mutation causes an alteration in auxin distribution, we introduced the auxin-responsive DR5::GUS construct into the null *ataucsia*-1 mutant (SALK_117986). Histochemical analyses of 5 day-old seedlings revealed differences in GUS expression between *ataucsia*-1 and wild-type plants. The DR5::GUS staining in wild-type primary root tip and central cylinder was stronger than that in *ataucsia*-1 mutant (Figure 3D). In the root apex, the *AtAucsia*-1 mutation resulted in a small reduction in DR5::GUS signal in a region of the stele just basal to the auxin apical maximum (Figure 3D). The pattern of DR5::GUS activity in *ataucsia*-1 mutant is consistent with an altered auxin distribution or sensitivity. Altogether these results indicate that inactivation of *AtAucsia*-1 gene causes an altered transport of auxin. The reduced steady state level of transcripts coding for ABCB auxin transporters is consistent with a reduction of PAT.

### *ataucsia*-1 Roots Have an Altered Response to Exogenous Auxin

**Figure 4:**
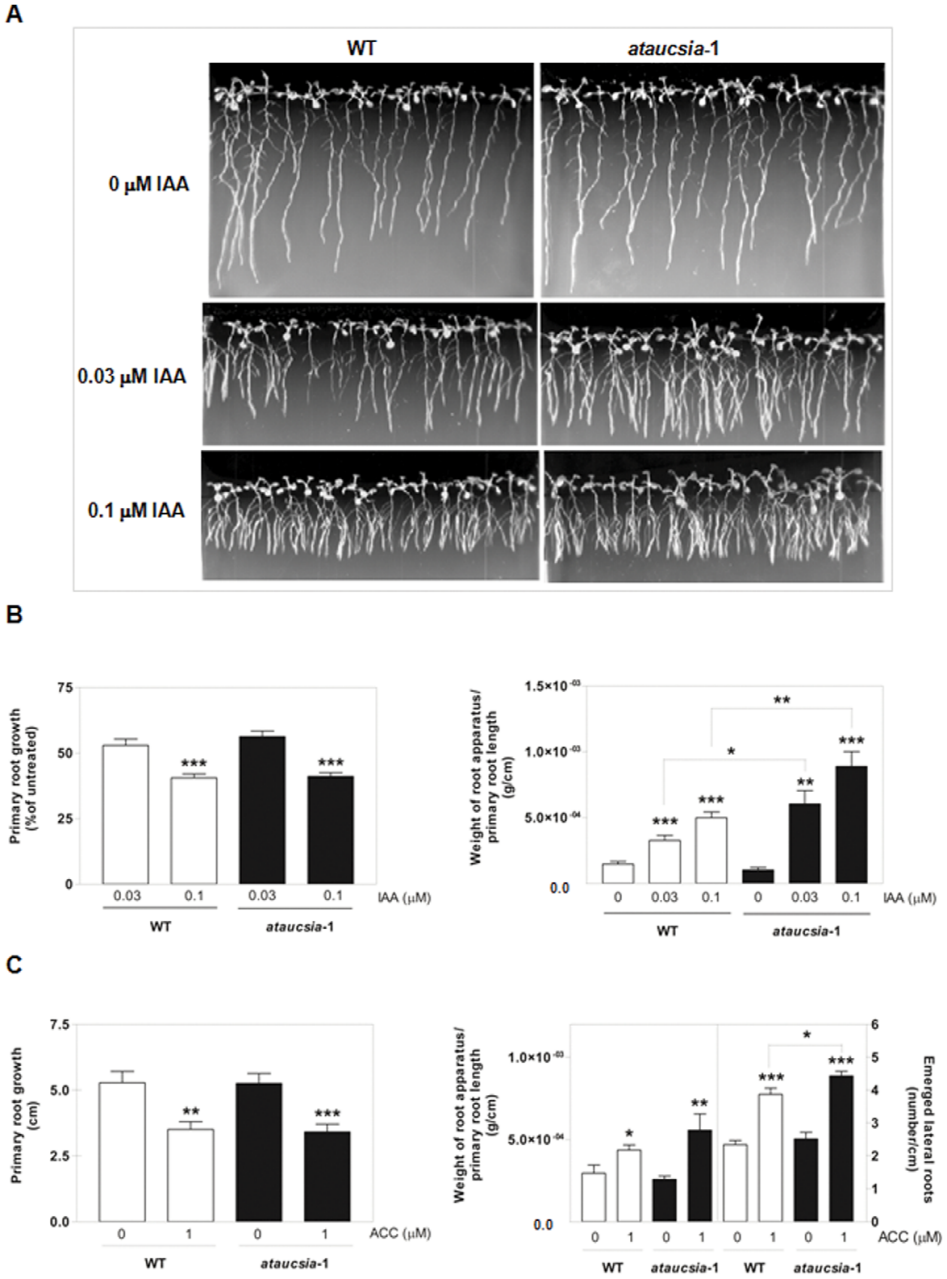
Effects of exogenous IAA and ACC on *ataucsia*-1 root growth. (A) Root phenotype of 10 days old wild-type and *ataucsia*-1 seedlings after treatment with IAA (0.03 and 0.1 µM). (B) Quantification of IAA effect on root growth. Seeds of wild-type and *ataucsia*-1 were germinated on nutrient medium for 5 days and then transferred to new medium either supplemented or not supplemented with IAA (0, 0.03 and 0.1 µM). Five days later, new root growth was evaluated. (Left) New primary root growth was measured and plotted as percentage of root growth on medium without IAA. (Right) Weight of root apparatus per cm of primary root length. (C) Quantification of ACC effect on root growth. As performed for panel B, 5 days old seedlings were transferred to medium supplemented or not with ACC (0, and 1 µM). Five days later, root growth was evaluated. (Left) Primary root growth. (Right) Two sets of y-axes are reported, on the left y-axis the weight of root apparatus per cm of primary root length and on the right y-axis the density of emerged lateral roots (number of lateral roots per cm of primary root). The values reported in B and C are means ± SE (n = 3) 15–20 seedlings per replicate. Student's *t*-test was applied. *P<0.05; **P<0.01; ***P<0.001 versus untreated seedlings or versus treated wild-type seedlings when indicated by brackets.

Both acropetal and basipetal auxin transport systems are crucial for lateral root development [40]. Since *ataucsia*-1 mutants are impaired in PAT as compared with wild-type, we investigated the effect of *ataucsia*-1 mutation on primary and lateral root growth in the presence or absence of exogenous auxin. Primary root growth and lateral root initiation and elongation can be stimulated or inhibited by exogenous auxin depending on the concentration [10], [41]. 5-day-old seedlings were treated with 0.03 and 0.1 µM indole-3-acetic acid (IAA). As already reported [41], these concentrations had inhibitory effects on primary root growth. The effect of IAA on primary root growth was similar in *ataucsia*-1 mutant (SALK_ 117986) and wild-type seedlings (Figure 4A and 4B). At the IAA concentrations tested, lateral root growth (evaluated as weight of root apparatus per cm of primary root length [42]) was enhanced in wild-type plants (Figure 4B), but *ataucsia*-1 mutant displayed a more pronounced increase in lateral root growth at both 0.03 and 0.1 µM IAA concentrations (Figure 4B). On the other hand, the lateral root density (calculated as number of lateral roots per cm of primary root excluding the distal region not forming the lateral roots) in auxin-treated *ataucsia*-1 mutant and wild-type seedlings did not differ. These data indicate that the promoting effect of IAA on lateral root growth is enhanced in *ataucsia*-1 mutant, whereas auxin-regulated lateral root initiation is unaffected.

It is well known that lateral root development in *A. thaliana* is controlled, among other signal interactions, by crosstalk between auxin and ethylene [43], [44], [45]. Recently, the role of ethylene in modulating auxin transport has emerged as an important aspect of the complex interactions between these two phytohormones [45], [46]. Along with the well-characterised reduction in primary root elongation [46], application of a high concentration (1 µM) of 1-aminocyclopropane-1-carboxylic acid (ACC), the ethylene precursor, negatively regulates lateral root initiation but it promotes the emergence and the elongation of existing lateral root primordia [47]. At 1 µM ACC, primary root growth of both wild-type and *ataucsia*-1 mutant (SALK_ 117986) plants were similarly inhibited (Figure 4C). However, *ataucsia*-1 seedlings showed a slightly enhanced sensitivity to the effect of ethylene on growth and density of lateral roots (Figure 4C), indicating that the response of *ataucsia*-1 mutant seedlings to ethylene is increased.

Inhibition of IAA polar transport by IAA efflux inhibitors, such as naphthylphthalamic acid (1-NPA), results in a variety of phenotypes including defects at the root level [48]. Considering alterations in PAT observed in *ataucsia*-1 mutant roots we investigated the sensitivity of *ataucsia*-1 mutant (SALK_ 117986) to 1-NPA (Figure S2). The inhibitory effect of 0.1 µM 1-NPA on density of emerged lateral roots was more evident in *ataucsia*-1 than in wild-type seedlings (Figure S2A). At higher concentration (0.5 µM 1-NPA) lateral root formation was almost completely suppressed in both *ataucsia*-1 mutant and wild-type seedlings (Figure S2A). Furthermore, the growth of the *ataucsia*-1 mutant primary root was less sensitive to 30 µM 1-NPA than those of wild type (Figure S2B).

### *AtAucsia*-1 Overexpressing Plants Display a Reduced Primary Root Length

**Figure 5:**
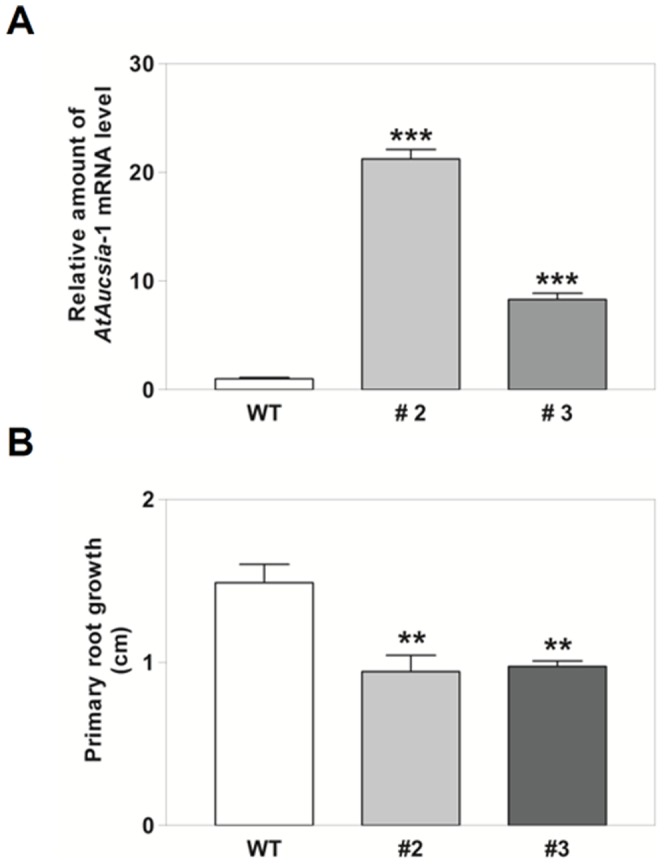
Overexpression of *AtAucsia*-1 gene in *A.thaliana*. (A) Quantitative real-time PCR analysis of *AtAucsia*-1 gene expression in wild-type, AtAucsia-1OX #2 and AtAucsia-1OX #3 lines transformed with Pro35S::AtAucsia-1 construct. Mean expression levels ± SE are shown. (B) Primary root growth of wild-type and AtAucsia-1OX #2 and AtAucsia-1OX #3 lines evaluated in 5 days old seedlings. Mean expression levels ± SE are reported, (n = 3) 15–20 seedlings per replicate. Data reported were analyzed by Student's *t*-test. **P<0.01; ***P<0.001 versus wild-type seedlings.

To further elucidate the biological role of *AtAucsia*-1, we analyzed *A. thaliana* plants that constitutively overexpress *AtAucsia*-1. The 162-bp long cDNA sequence corresponding to the coding region of *AtAucsia*-1 was introduced under the control of the constitutive CaMV 35S promoter (Pro_35S_:AtAucsia-1) in wild-type *A. thaliana* plants. Two lines (hereafter referred to as AtAucsia-1OX #2 and #3) among the 4 independent lines generated, were used for subsequent analyses. AtAucsia-1OX #2 line showed the highest level of expression, i.e. approximately twentyfold higher *AtAucsia*-1 mRNA level than wild-type, whilst AtAucsia-1OX #3 line expressed eightfold higher level than wild-type (Figure 5A). Analysis of 5 days old AtAucsia-1OX T3 progenies of both lines, revealed an approximately 35% reduction of primary root growth in comparison to wild-type (Figure 5B). This result indicates a role for the *AtAucsia*-1 gene in root growth. To test whether auxin response was altered in AtAucsia-1OX roots, AtAucsia-1OX #2 line was grown in the presence of exogenous IAA. As reported in figure S3A, *AtAucsia*-1 overexpression determines a decreased sensitivity to the inhibitory effect of IAA (0.03 and 0.1 µM) on primary root elongation, but did not affect the lateral root density (data not shown).

Overexpression of *AtAucsia*-1 did result in visible alterations in reproductive development only in the strongest overexpressing line (AtAucsia-1OX #2) (Figure S3B). AtAucsia-1OX #2 flowers showed a reduced length of stamen filament, stamen filament fusions and abnormal stamen orientation (Figure S3B). AtAucsia-1OX #2 plants produced a reduced number of seeds. The low fertility can be most likely explained by inefficient delivery of pollen caused by reduced filament length, consistent with previous reports of a role for auxin in filament elongation [49], [50].

### Analysis of *AtAucsia*-1 Expression Pattern

**Figure 6:**
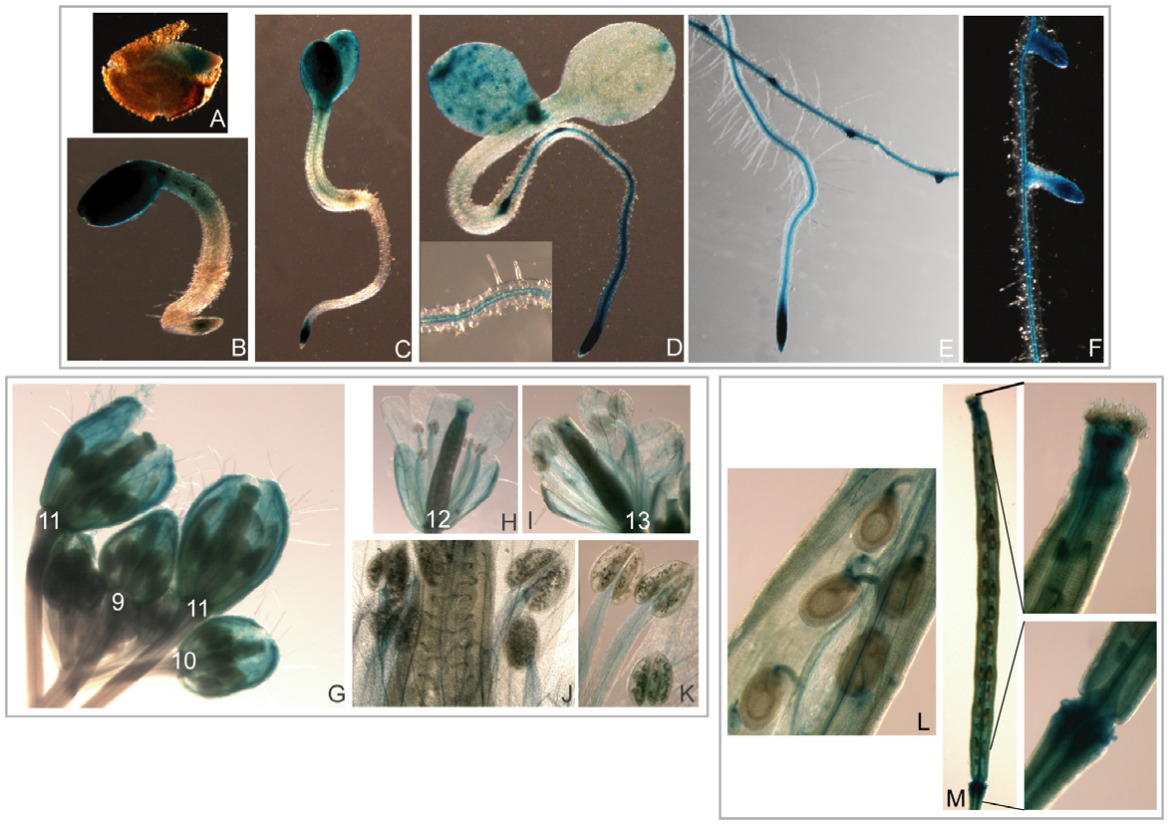
Histochemical analysis of GUS activity driven by the *AtAucsia*-1 promoter in *A. thaliana*. (A–D) GUS activity at different stages of germination. (A) embryo at mature cotyledon stage; (B) seedling 1 day after germination (dag); (C) 3 dag seedling, (D) 4 dag seedling, a particular of the primary root vasculature is reported in the inset; (E) and (F) 10 days old seedling, GUS staining patterns in emerged lateral roots. (G) stages 9–11 of flower development (for a description of the stages see results and [75]. (H–I) stages 12–13 of flower development; (J–K) stage 12–13, magnifications highlighting GUS expression in anther filaments. (L–M) growing silique approximately 1 cm long. (L) GUS expression in ovule funiculi, (M) GUS activity in the silique.

To monitor *AtAucsia*-1 expression pattern in developing roots and flower organs, promoter-reporter gene fusion analysis was performed. Wild-type plants were transformed with Pro_AtAucsia-1_::GUS, containing as promoter the 781 bp-long DNA sequence upstream the ATG initiation codon (Figure S4A), fused to a β-glucuronidase (GUS) reporter gene. Several *A. thaliana* lines transgenic for Pro_AtAucsia-1_::GUS gene construct were histochemically stained with 5-bromo-4-chloro-3-indolyl- β-glucuronic acid (X-Gluc) at different developmental stages and representative expression patterns are shown in figure 6. Analysis of Pro_AtAucsia-1_::GUS activity revealed that *AtAucsia*-1 is expressed both in mature seeds and seedlings. In seeds at the mature cotyledon stage, the highest level of expression has been observed in the radicle (Figure 6A). In seedlings (1–4 days after germination), Pro_AtAucsia-1_::GUS was mainly expressed in cotyledons and at the tip of primary roots (Figure 6B and 6C). At subsequent stages (5–7 days after germination), *AtAucsia*-1 expression was visible in the primary root both in the vascular tissue and at the root tip (Figure 6D). *AtAucsia*-1 gene expression was detectable also in the emerging and in the entire lateral root (Figure 6E and 6F). GUS activity was also examined during stages 9–13 of floral development. At stages 9–11, GUS activity was mainly detected in sepal's vasculature and in the stigma (Figure 6G). At stage 12 and 13 (i.e. anthesis), the major sites of GUS staining were the stigma, vascular tissues of petals and sepals, stamen filaments and the vascular tissue of the ovule funiculus (Figure 6H, 6I, 6J, and 6K). In growing siliques, GUS activity was evident in the ovule funiculi, in the septum, in the pedicel and at the apical tip of the siliques (Figure 6L and 6M). The *AtAucsia*-1 expression pattern is different, at least in some organs and tissues, from that observed in *A. thaliana* plants transformed with a construct containing the GUS coding region driven by *AtAucsia*-2 promoter consisting of 1246 bp-long DNA sequence upstream the ATG initiation codon (Pro_AtAucsia-2_::GUS) (Figure S4B and S4C). *AtAucsia*-2 expression was very strong in cotyledons and hypocotyls of young seedlings, and within the root (5–7 days after germination), it was preferentially expressed at the apex of primary and lateral roots (Figure S4C). In the female gametophyte, *AtAucsia*-2 gene displayed an overlapping expression with *AtAucsia*-1 gene in the vascular tissues of the carpel and in the vasculature of the ovule funiculus (Figure S4C). However, in male reproductive organs, *AtAucsia*-2 expression is confined to anther and mature pollen grain (Figure S4C).

It is important to mention that RNAi of both *Aucsia* genes causes parthenocarpic development of the tomato fruit [1]. Taking into consideration the expression pattern of *AtAucsia* genes in female reproductive organs (i.e ovule funiculus), flowers of the *ataucsia*-1 mutant (SALK_117986) were emasculated to test for parthenocarpy. Silique development in emasculated *ataucsia*-1 mutant flowers was compared with those of wild-type emasculated flowers. Emasculation of 40 flowers from *ataucsia*-1 plants produced pistils that did not significantly elongate compared with those derived from wild-type flowers, indicating that knock-out of *AtAucsia*-1 function does not cause parthenocarpic development of the silique (data not shown).

### AtAUCSIA-1 Interacts with a Kinesin-related Protein

**Figure 7:**
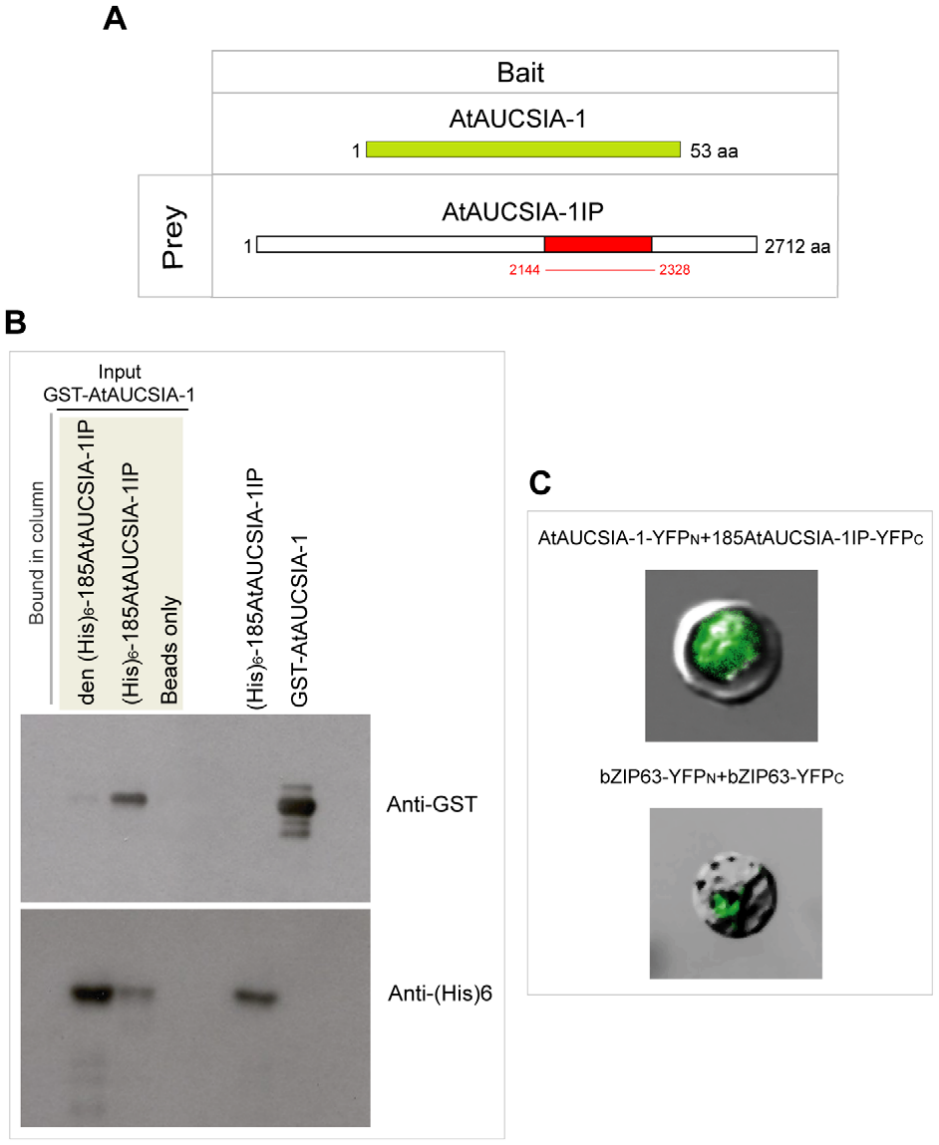
AtAUCSIA-1 interacts with a kinesis-related protein (AtAUCSIA-1IP; i.d. Q9SB74). (A) AtAUCSIA-1IP protein was identified by yeast two-hybrid screening. Five independent clones, corresponding to portions of AtAUCSIA-1IP protein, interacted with the entire AtAUCSIA-1 protein (green box) defining a common overlapping domain of 185 amino acids (red box, 185AtAUCSIA-1IP). (B) The GST-AUCSIA-1 fusion protein interacts with (His)_6_-185AtAUCSIA-1IP in an *in vitro* binding assay. (His)_6_-185AtAUCSIA-1IP proteins, either in denatured (den (His)_6_-185AtAUCSIA-1IP den) or native form ((His)_6_-185AtAUCSIA-1IP), were bound to nickel-nitrilotriacetic acid magnetic beads. Then, the magnetic beads were incubated in the presence of purified GST-AUCSIA-1. The protein fractions bound to magnetic beads were eluted, separated by SDS-PAGE, and detected by Western blot using either anti-GST (upper panel) or anti-His antibodies (lower panel). (His)_6_-185AtAUCSIA-1IP coated beads pulled down GST-AUCSIA-1. Beads alone were used as negative control to test non specific interactions. Purified (His)_6_-185AtAUCSIA-1IP and GST-AUCSIA-1 proteins were used as protein markers in SDS-PAGE. (C) BiFC experiments were performed in *A. thaliana* mesophyll protoplasts by coexpression of translational fusions of AtAUCSIA-1 and 185AtAUCSIA-1IP with either the C- or N-terminal halves of YFP (YFP_C_ or YFP_N_). AtAUCSIA-1 (AtAUCSIA-1-YFP_N_) interacts with 185AtAUCSIA-1IP (185AtAUCSIA-1IP-YFP_C_) protein in the cytoplasm (upper panel). An identical result was obtained by swapping the two YFP portions (data not shown). For positive control, the *A.thaliana* leucine zipper bZIP63 fused to YFP_N_ and YFP_C_ was used (lower panel) [102]. The dimer of bZIP63 was located in the nucleus (lower). The fluorescence signals were detected by a confocal laser scanning microscope (Fluoview FV1000; Olympus).

To further investigate the functional role of AtAUCSIA-1, its putative interacting partners were screened by yeast two-hybrid (Y2H) system [51]. The bait vector contained the full length *AtAucsia*-1 sequence cloned downstream the LexA DNA Binding Domain, whilst the prey vectors contained the cDNA sequences, representing the whole cDNA library obtained from 7-day-old *A. thaliana* seedlings, fused in frame with the Activation Domain of Gal4. The screening of this library led to the identification of an interacting protein fragment belonging to a larger protein (hereafter named AtAUCSIA-1IP) with a high biological score amongst the different putative interacting protein fragments (see Material and Methods for details). AtAUCSIA-1IP is encoded by the 8136 bp-long ORF of the *At4g31570* gene. The prey fragment, 185 amino acids long, correspond to the region from amino acid 2144 to amino acid 2328 of the entire protein, that is 2712 amino acids long (Figure 7A). The interaction between AtAUCSIA-1 and the protein fragment of AtAUCSIA-1IP was confirmed performing an *in vitro* pull-down assay (Figure 7B). The coding sequence of *AtAucsia*-1 was cloned into pGEX4T1 expression vector, to produce a glutathione *S*-transferase (GST) fusion protein, GST-AtAUCSIA-1. The sequence corresponding to the 185 amino acids long portion of AtAUCSIA-1IP, was cloned into pET12b expression vector to produce a (His)_6_-tagged protein ((His)_6_-185AtAUCSIA-1IP). Purified recombinant proteins were used for *in vitro* binding experiment, immobilizing (His)_6_-185AtAUCSIA-1IP on nickel coupled agarose beads. As reported in figure 7B, a direct interaction between the recombinant GST-AtAUCSIA-1 and (His)_6_-185AtAUCSIA-1IP was verified by using antiGST monoclonal antibody. To determine whether AtAUCSIA-1 interacts with AtAUCSIA-1IP-derived protein fragment *in vivo*, a bimolecular fluorescence complementation (BiFC) system was used. Translational fusion constructs of AtAUCSIA-1 and 185AtAUCSIA-1IP with either the C-terminal or the N-terminal half of the Yellow Fluorescent Protein (YFP) were co-transfected in *A. thaliana* protoplasts. This analysis has shown that AtAUCSIA-1 interacts with the identified 185 amino acids long fragment of AtAUCSIA-1IP in *Arabidopsis* and revealed that the interaction between the two partners occurs in the cytoplasm (Figure 7C, upper panel).

**Figure 8:**
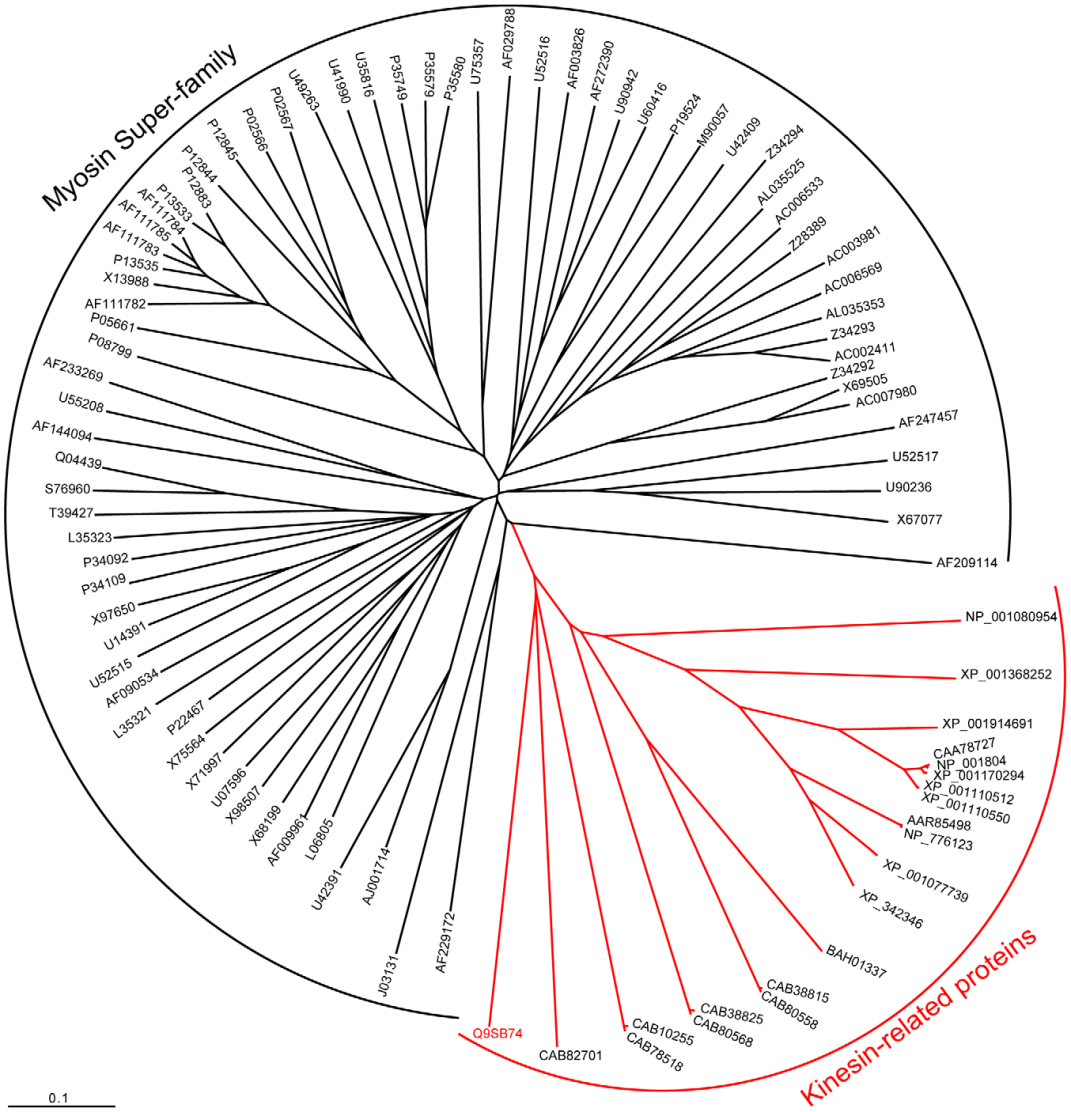
Unrooted Phylogenetic tree. Representative members of the myosin superfamily [53] and kinesin-like proteins were aligned with *A. thaliana* AtAUCSIA-1IP (i.e Q9SB74, written in red) and a phylogenetic tree was built exploiting the Clustal W algorithm [103]. The protein sequences used in the phylogenetic analysis are listed as follows. For myosin super-family: *Homo sapiens* (AF009961, X68199, X98507, X71997, U14391, X97650, AF111785, AF111784, X13988, AF111783, P13533, P12883, P13535, P35579, P35580, P35749, AF111782, AF229172, U90942, U60416, AF272390, U90236, U55208, AJ001714, U42391, AF247457, AF144094, AF209114), *Drosophila melanogaster* (U07596, P05661, U35816, J03131, AF003826, X67077, AF233269), *Caenorhabditis elegans* (X75564, U52515, P12844, P02566, P12845, P02567, U41990, U49263, U52516, U52517), *Saccharomyces cerevisiae* (S76960, Q04439, P19524, M90057), *Schizosaccharomyces pombe* (T39427, U75357, AF029788), *Dictyostelium discoideum* (P22467, P34092, L35323, P34109, L06805, AF090534, P08799, L35321, U42409), *Arabidopsis thaliana* (X69505, Z34292, AC007980, Z28389, Z34293, Z34294, AL035525, AC006569, AL035353, AC002411, AC006533, AC003981). For kinesin-related proteins: *H. sapiens* (CAA78727, NP_001804), *Xenopus laevis* (NP_001080954), *Mus musculus* (NP_776123, AAR85498), *Equus caballus* (XP_001914691), *Monodelphis domestica* (XP_001368252), *Pan troglodytes* (XP_001170294), *Rattus norvegicus* (XP_001077739, XP_342346), *Macaca mulatta* (XP_001110550, XP_001110512), *Arabidopsis thaliana* (CAB78518, CAB10255, CAB82701, CAB80568, CAB80558, CAB38825, CAB38815), *Oryza sativa* (BAH01337). Bar represents 0.1 point mutation per sequence position.

*At4g31570* is annotated as a gene of unknown function, and yet by using the tblastx algorithm [52] it was found that *At4g31570* contains a region of homology with a myosin-like protein of *Oryza sativa* (AY224554). Taking this putative similarity into account, the predicted protein sequence of AtAUCSIA-1IP (i.d. Q9SB74) was aligned with 72 representative members of the myosin superfamily from different organisms (i.e. mammals, *Drosophila*, *Dictyostelium*, *Caenorhabditis elegans*, *A. thaliana*, *Saccharomyces cerevisiae* and *Schizosaccharomyces pombe*) described by Berg and collaborators [53]. Contrary to expectations, the phylogenetic analysis showed that AtAUCSIA-1IP clusters with some members of human-derived myosins and not with those of *A. thaliana* (data not shown). The analysis of AtAUCSIA-1IP by NCBI's conserved domain database (CDD) revealed the presence of two repeats of the chromosome segregation ATPase domain, commonly found in proteins involved in cell division and chromosome partitioning belonging to the kinesin-related group of proteins. A new phylogenetic analysis, which included members of both myosin and kinesin superfamilies, was performed in order to clarify which class of protein AtAUCSIA-1IP resembles most. AtAUCSIA-1IP clearly clusterized with kinesin-related centromere proteins E (CENP-E) (Figure 8).

To tentatively assign a function to AtAUCSIA-1 and to AtAUCSIA-1IP, we have also adopted a recent bioinformatic tool for gene ontology prediction in *A.thaliana* (GO-At tool (http://www.bioinformatics.leeds.ac.uk/goat/) [54]. Using GO-At, a list of genes ranked in descending order of probability of functional association with *At4g31570* was generated. Taking the biological function with the best prediction score, AtAUCSIA-1IP is described as a protein involved in “actin filament-based movement”, while AtAUCSIA-1 is predicted to be involved in “microtubule-based movement”. Thus, the GO-AT analysis indicates an involvement of both AtAUCSIA-1 and AtAUCSIA-1IP in intracellular movements based on cytoskeleton structural components.

The Y2H screening has identified other putative partners. Amongst candidates with a lower biological score compared to AtAUCSIA-1IP, another putative AtAUCSIA-1 interacting protein is a 1-aminocyclopropane-1-carboxylate oxidase (ACO2; *At1g62380*) that catalyses the final step of ethylene synthesis. The prey fragment consists of the first N-terminal 148 amino acids (Figure S5A). The interaction has been confirmed *in vitro* by using pull-down assay (Figure S5B). BIFC assays for this and other candidates are obviously necessary to validate the interaction *in vivo*. Nevertheless, it is perhaps relevant to mention that amongst the Y2H candidates with score equal to AtAUCSIA-1IP, an Ankyrin repeat protein (*At4g35450*), an EF Calcium binding protein (*At4g38810*) and PROTEOLYSIS 6 (PRT6) (*At5g02310*) were found. PRT6 is an UBR ubiquitin ligase able to mediate degradation of proteins with an amino-terminal arginine residue [55]. PRT6 belongs to the same gene family of BIG, a protein involved in the control of auxin transport [56]. The PRT6 prey fragment interacting with AtAUCSIA-1 spanning amino acids 20-498 includes the UBR domain.

## Discussion

*Aucsia* genes have been discovered and functionally identified in tomato by RNA interference of both *SlAucsia*-1 and *SlAucsia*-2 [1]. *Aucsia* is a green plant gene family that encodes miniproteins 44–54 amino acids long characterized by a conserved 16 amino acids long motif. The high degree of conservation of AUCSIA miniproteins suggests that *Aucsia* genes should have similar functions, at least in plants belonging to the angiosperms. In this work, we have shown that *AtAucsia*-1 (*At3g01130*) operates by itself in the auxin biology of the root. Y2H, BIFC assays and pull-down experiments have shown that the AtAUCSIA-1 interacts with a CENP-E kinesin-related protein encoded by the *At4g31570* locus.

In angiosperms, auxin biology stands mainly on synthesis, homeostasis, transport and signal transduction [4], [5], [57]. During land plants evolution, biological complexity could have arisen by expansion of functional modules in each one of the four forementioned mechanisms, via duplication and neo-sub-functionalization of genes/proteins [58], [59] and/or via an increased combination of molecular components, i.e. increasing the molecular connectivity of the system [60], [61].

Angiosperms bear flowers and fruits, the last innovation of plant evolution. The floral organs are modified leaves and auxin-based mechanisms, acting in leaves and other lateral organs, have been recruited with modifications in the development of the gynoecium and in fruit initiation [62], [63]. Fruit initiation usually takes place only after pollination and fertilization of the ovary. Auxin, either exogenously supplied [64] or endogenously synthesized in the placenta-ovules [65], initiates fruit development in the absence of pollination/fertilization (i.e. parthenocarpy). In tomato, RNA interference of both *Aucsia* genes causes parthenocarpy, reduces polar auxin transport (PAT) in the root, alters leaf vascular development and curtails auxin-induced rhizogenesis [1]. *Aucsia* genes are present in both land plants and Chlorophyta (*Ostreococcus*, *Helicosporidium*, *Prototheca*, *Chlorella*, and *Coccomyxa*). Consequently, they were already present before the divergence of Chlorophyta and Streptophyta, and yet they control fruit initiation, an innovation likely not much older than 160 millions of years. The knock-out of *AtAucsia*-1 gene expression (i.e. *ataucsia*-1 null mutant) did not cause parthenocarpy. Thus, either *Aucsia* genes have overlapping expression patterns and roles in fruit development and consequently *AtAucsia*-2 compensates for *AtAucsia*-1 loss of function, or this function can only be attributed to *AtAucsia*-2. Moreover, although many molecular mechanisms controlling fruit development are conserved in dry and fleshy fruits [66], the data presented do not allow to exclude the possibility that the described role of *Aucsia* family in tomato fruit initiation is restricted to fleshy fruits.

Homozygous plants for a T-DNA insertion (SALK_065659) in the 5′ULR of *AtAucsia*-2 (*At5g15320*) express approximately 50% of *AtAucsia*-2 transcript level in comparison to wild-type. We were not able to get homozygous seeds of another T-DNA insertion (SALK_097153) in the promoter region of *AtAucsia*-2. Thus, to dissect the biological role of *AtAucsia*-1 from that of *AtAucsia*-2, we have characterised their expression patterns by expressing the GUS reporter under the control of either the *AtAucsia*-1 or the *AtAucsia*-2 promoter. The two *AtAucsia* genes are ubiquitously expressed in both vegetative and reproductive organs. In young seedlings (1–5 days after germination) both *AtAucsia* genes are preferentially expressed at the tips of primary and lateral roots. However, in older seedlings (5–7 days after germination), *AtAucsia*-1 is strongly expressed also in the root vasculature. The expression of *AtAucsia*-1 in the root vasculature is a prerogative of the *AtAucsia*-1 gene. Differences in gene expression were detected in the stamens, too. *AtAucsia*-1 was expressed in the anther filament, whereas *AtAucsia*-2 was preferentially expressed in the anther and pollen grains. Thus, the gene expression analysis has shown that transcript distribution of the two *Aucsia* genes, at least in the root and the stamen, is not completely overlapping. Consequently, a null *AtAucsia*-1 mutation might cause phenotypical alterations detectable in the root and/or in the stamen. In this regard, *Aucsia*-silenced tomato plants have shown that most, although not all auxin-related phenotypic alterations concern the root apparatus and flower organs [1].

The loss of *AtAucsia*-1 function alters auxin biology in the root. In *ataucsia*-1 mutants' roots polar auxin transport (PAT), both acropetal and basipetal, was reduced by approximately 30% as compared with wild-type plants. PAT supplies the auxin that is needed for lateral root initiation and elongation [67] and mutants altered in auxin biology often show lateral roots defects and/or altered sensitivity to exogenous auxin [68], [69]. The *ataucsia*-1 mutant was more sensitive to the promoting effect of exogenous auxin on lateral root growth as compared with wild type. Similarly, *ataucsia*-1 displayed a higher responsiveness to an ethylene concentration that stimulates lateral root growth. Ethylene and auxin interact to regulate the initiation, emergence and elongation of lateral roots [47], [70]. One mode of action of ethylene in regulating root growth is the modulation of polar auxin transport along with a positive effect on auxin synthesis [45]. Ethylene enhances both the acropetal and basipetal auxin transport, and affects lateral auxin flow [45]. The altered root growth responses of *ataucsia*-1 mutants to exogenous auxin and ethylene could be the result of an anomalous distribution of auxin as a consequence mainly of the impaired basipetal and acropetal polar auxin transport.

Exogenously applied auxin efflux inhibitor N-1-naphthylphtalamic acid (1-NPA) can determine either an arrest of lateral root development or inhibition of primary root growth depending on the concentrations [10]. The altered 1-NPA sensitivity displayed by *ataucsia*-1 mutants further supports the finding that the mutation affects auxin transport. Overall, these results indicate that *AtAucsia*-1 is required for long distance PAT and consequently contribute to auxin-mediated root development control. In plants overexpressing *AtAucsia*-1, the growth of the primary root is impaired and showed an altered sensitivity to the inhibitory effect of IAA on primary root growth. This finding is consistent with the hypothesis that *AtAucsia*-1 functions as a regulator of auxin transport and that it is involved in auxin biology. The growth inhibition of primary root might be caused by an increase in long distance PAT from the shoots to the roots causing an altered distribution of auxin in the root apex. However, a similar effect might be caused by different mechanisms affecting auxin biology. For example, a shorter root phenotype has been observed in activation-tagged *yucca* mutants that displayed an increased auxin levels in the roots [71], [72], [73]. Yucca genes code for flavin monooxygenase-like (FMO) enzymes involved in auxin biosynthesis [71], [74].

The flowers of *ataucsia*-1 null mutant did not show visible morphological changes, whereas in the AtAucsia-1OX#2 overexpressing line (i.e. the strong overexpressing line analysed) the majority of flowers at anthesis contained stamens with reduced filament length. It is well known that auxin acts at the anther-filament junction to promote pre-anthesis filament growth and that auxin transport from the anther to the filament is relevant for filament elongation [75]. A short filament phenotype has been observed in *abcb19abcb1* double mutant defective in the auxin transporters ABCB19 and ABCB1 [76]. In *ataucsia*-1 mutant the RNA steady state levels of *ABCB1*, *ABCB4* and *ABCB19* were reduced by approximately 30% in comparison with wild type, whereas *PIN1*, *PIN2*, *PIN 3* and *PIN7* levels were unaffected. The steady state levels of the *ABCG36* and *ABCG37* transcripts, two IBA transporters, were unaffected too. Transcript level of *AUX1*, an IAA symporter, was also unchanged (Data not shown). *ABCB1*, *ABCB4* and *ABCB19* are known to have different spatial expression patterns in the roots. ABCB19 and ABCB1 operate in long-distance auxin transport, whereas ABCB4 functions in the movement of auxin away from the root tip. Thus, a reduced expression level of the three transporters is in accordance with the observations that both acropetal and basipetal PAT is impaired in *ataucsia*-1 mutant. However, the present knowledge cannot establish a direct causative link between the reduced auxin transport in *ataucsia*-1 mutant and the decreased expression of these ABCBs auxin transporters. Expression of *ABCB1*, *ABCB4*, and *ABCB19* is up-regulated by auxin [19], [69], [76], [77] and changes in their gene expression levels appear to mirror alteration in the protein abundances [15], [19], [69], [77], [78]. ABCB auxin exporters appear to be particularly relevant in rapidly growing tissues and in long distance PAT, while PIN plasmamembrane transporters act specifically to control PAT directionality [5]. The observation that the expression of the DR5::GUS reporter gene [79] in *ataucsia*-1 null mutants showed a reduced intensity of GUS staining at the tip of the primary root in comparison to wild-type plants indicates that auxin activity and consequently auxin content in the root apex was reduced.

PAT depends on specific auxin influx and efflux transport proteins localized at the plasma membrane, and yet another layer of complexity is represented by the role of cytoskeleton in the regulation of auxin transporters' trafficking [80]. The activity and polarization of auxin transporters is brought about through different mechanisms such as endocytosis, endosomal sorting and recycling, and transcytosis [80]. These endomembrane trafficking processes are intimately linked to the dynamics of microfilaments and microtubules [80]. Experimental evidence has already suggested that the distribution of auxin transporters, such as AUX1 and PINs, is affected by cytoskeleton dynamics [80], [81], [82]. For instance, some inhibitors of PAT act through actin remodelling [83], [84]. In addition, auxin-mediated regulation of actin structure is considered to be one of the mechanisms involved in the autoregulatory loop of auxin flow [85]. A role of endocytosis and cytoskeleton dynamics is also part of ABP1-dependent auxin signal transduction pathways [35], [36]. Thus, several aspects of auxin biology are based on cytoskeleton-based processes.

A crucial aspect of this work is the identification of AtAUCSIA-1IP, a rather large protein physically interacting with AtAUCSIA-1. AtAUCSIA-1IP function is unknown and no obvious morphological differences were observed in its knock-out T-DNA insertion mutant (data not shown). Bioinformatics analysis has revealed that AtAUCSIA-1IP shows homology and clusters with CENP-E kinesin-related proteins. Kinesins are motor proteins interacting with microtubules [86], and yet some kinesin-like proteins can interact directly with actin filaments [87], [88], [89], [90]. Moreover some plant kinesins, i.e. kinesins with Calponin-homology domain, have been associated with a putative role in microtubule-microfilaments crosslinking [91]. Furthermore, according to the GO-At web tool [54] for *in silico* prediction of gene function, AtAUCSIA-1IP is described as a protein involved in “actin filament-based movement”. The same analysis predicts for AtAUCSIA-1 protein a role in microtubule-based process. Considering that AtAUCSIA-1 is involved in auxin biology and auxin transport and that both AtAUCSIA-1 and AtAUCSIA-1IP are predicted to be cytoskeleton associated proteins, the participation of the AtAUCSIA-1/AtAUCSIA-1IP complex in the cytoskeleton regulation of intracellular trafficking can be proposed.

Due to its minimal molecular mass, AUCSIA is a likely regulatory component of multiprotein complex(es). AUCSIA miniproteins conserved features allow envisaging several possible regulatory mechanisms. First, AUCSIA miniproteins contain a tyrosine-based endocytic motif. Tyrosine-based endocytic motifs bind Adaptor Protein complexes and are involved in clathrin-mediated endocytosis [92], [93]. A second interesting feature is that the first serine residue of the AUCSIA motif (PYSGXStLAlvaRXsA) is a candidate for alternate phosphorylation/O-N-acetylglucosamine glycosylation (YinOYang program) [94]. AUCSIA might be either phoshorylated or O-GlcNAc glycosylated at the same serine residue of the AUCSIA motif. O-GlcNAcylation, like phosphorylation and ubiquitination, predates the divergence of Eukarya and in multicellular organisms it represents a molecular feature regulating multi-component complexes [95].

In different green plant, AUCSIA carboxyterminal region ranges between 10 and 16 amino acid residues, shows no conserved sequence, and yet it is always rich in lysine residues. Lysine residues are possible sites of ubiquitination. Monoubiquitination of several lysine residues of the same protein or polyubiquitination via lysine K63 are signals for endocytosis, whilst polyubiquitination of ubiquitin via lysine K48 targets the protein to proteasome degradation [96], [97]. Phosphorylated or O-GlcNAc glycosylated AUCSIA miniproteins might interact with different molecular partners increasing the molecular connectivity of auxin biology. In vascular plants, connections between auxin synthesis and PAT have to exist in order to solve the need of either increasing auxin flow from localized territories of auxin synthesis or decreasing auxin flow from a source in response to changes in the nutritional status of the plant and/or plant organs.

## Materials and Methods

### Plant Material and Growth Conditions

*Arabidopsis thaliana* ecotype Columbia (Col) was used. Seeds were surface-sterilised and *in vitro* germinated on MS agar plates (2.15 g/L MS salts, 0.8% plant agar (w/v), 1% sucrose, pH 5.7) as previously described [98]. The seedlings were grown under sterile conditions in a climatic chamber at a constant temperature of 25°C during a 10 h/14 h light/dark cycle, with an average irradiance of 120 µmol m^−2^ sec^−1^ of photosynthetically active radiation (PAR). The plates were maintained in darkness at 4°C for 2 days to induce even germination.

### GUS Constructs and Histochemical Staining

To create the transcriptional fusion construct Pro_AtAucsia-1_::GUS and Pro_AtAucsia-2_::GUS, 781 bp-long and 1246 bp-long sequences upstream of the *AtAucsia*-1 and *AtAucsia*-2 initiation codons, respectively, were amplified from wild-type plants via PCR with the following primers: for *AtAucsia*-1 promoter (F 5′-TCCTTCAAATCCTAATACGTTTT-3′ and R 5′-ATTAGCAGTTGAGATTAAACCC-3′) for *AtAucsia*-2 promoter (F 5′-AACTGTTCCATGCAAAATACT-3′ and R 5′-CAAATCACTGTTGGTCTCTC-3′). The PCR fragments were cloned into a derivative of pBin19 vector, which contains an 1812 bp-long sequence of GUS gene. The resulting chimeric genes were used to transform *Agrobacterium tumefaciens* strain GV2260. Pro_AtAucsia-1_::GUS and Pro_AtAucsia-2_::GUS gene constructs were introgressed in *A.thaliana* via genetic transformation using floral dipping [98]. The histochemical GUS staining was performed by submerging whole seedlings in GUS staining buffer containing 1 mM 5-bromo-4-chloro-3-indolyl β-D-glucuronidase, 100 mM sodium phosphate (pH 7.5), 0.5 mM potassium ferricyanide, 0.5 mM potassium ferrocyanide, 10 mM EDTA, and 0.1% (v/v) Triton X-100. Seedlings for all treatments were incubated at 37°C for 12–16 h and cleared with 70% (v/v) ethanol. Images were taken with Leica MZ16F stereomicroscope (Leica Microsystems).

### *AtAucsia*-1 T-DNA Insertional Mutants and Pro_35S_::*AtAucsia*-1 Overexpressing Lines

The Col plant lines SALK_117986 and SAIL_1146 were identified from the collection of SALK lines [37] and obtained from NASC (the European *Arabidopsis* Stock Centre). Selection plates were prepared adding to MS medium kanamycin at the final concentration of 50 mg L^−1^. Homozygous status was determined by PCR analysis following instructions of SIGnAL (Salk Institute Genomic Analysis Laboratory; http://signal.salk.edu/). *A. thaliana* plants (Col ecotype) were transformed with a derivative of pBin19 vector containing 162 bp-long sequence of *AtAucsia*-1 coding region, under the control of the CaMV 35S promoter by *Agrobacterium* GV2260-mediated floral dip method. Overexpression of *AtAucsia*-1 transgenic plants was confirmed by qRT-PCR analysis by using primers designed on the coding region.

### Quantitative RT-PCR

RNA was isolated using the RNeasy mini kit (QIAGEN) starting from 100 mg of frozen tissues and treated with RQ1 DNase (Promega). mRNA samples were checked for DNA contamination by performing PCR on the actin gene. Comparative PCR analysis was carried out using first strand cDNA obtained with oligo-(dT) primer and ImProm-II Reverse Transcription System (Promega). cDNA was amplified using SYBR Green qPCR Supermix-UDG (Invitrogen) on the ABI Prism 7000 Sequence Detection System (Applied Biosystems). The qRT-PCR was performed using the following cycling conditions: 2 min at 50°C, 2 min at 95°C, 40 cycles of 95°C for 30 sec, 56°C for 30 sec, 72°C for 30 sec and finally 72°C for 2 min. All quantifications were normalised to the actin gene as an endogenous control. For each determination of mRNA levels, three cDNA samples derived from three independent RNA extractions were analysed. Relative quantification of transcript levels was carried out following [99]. For *A. thaliana* qRT-PCR analysis, forward (F) and reverse (R) primers used for amplification of the target genes are the following: for *AtAucsia*-1 (F 5′-CTAATCAATGGCGCCACCTCCAGGA-3′ and R 5′-AGAGATAACAATCACTTGGCTTCATA-3′); for *AtAucsia*-2 (F 5′-TTTGACAATGACGCTACCTCCAGGT-3′ and R 5′-GAGACATCACCCCCAGCTTTTGG-3′); for *ABCB1* (F 5′-CCAGGCTTGCTCTGGTAGAACAT-3′ and R –GATTCCATCAGGATGGTTCTTG-3′), for *ABCB4* (F 5′-GTGTTATGGTAAACCGGACTACA-3′ and R 5′-ACTCCGTCTTTGATATTGATCAACG-3′), for *ABCB19* (F 5′-GAGGCTCATGAGAGGTCGGACCA-3′) and for actin (F 5′-TGTTCTCTCCTTGTACGCCAGT-3′ and R 5′-CAGCAAGGTCAAGACGGAGGA-3′).

### Phenotypic Analysis of *ataucsia*-1 homozygous Mutant and Pro_35S_::AtAucsia-1 Overexpressing Lines

For quantitative analysis of root growth, wild-type and mutant seeds were *in vitro* germinated in a vertical orientation. After 5 days, when the roots were 1.5 cm in length, the seedlings were transferred to a new plate containing either different IAA (0.03, and 0.1 µM) or ACC (1 µM) or 1-NPA (0.1, 0.5, 3, and 30 µM). Five days later, root growth was measured. For evaluation of the root gravity response, five days-old vertically grown seedlings were reoriented by 90°, and the angles of root curvature were measured at 2, 4, 6, 8, 24 and 48 h after reorientation by using ImageJ program (http://rsbweb.nih.gov/ij/). For evaluation of Pro_35S_:AtAucsia-1 overexpressing root growth, 5 days old seedlings were used, while for root response in the presence of exogenous IAA, seedlings were treated for a total of 10 days as described before.

For the scoring of *ataucsia*-1 parthenocarpy, 40 flowers were emasculated and pistils lengths were measured after 7 days and compared with those derived from wild-type emasculated flowers.

### Polar Auxin Transport Analysis in *A. thaliana* Roots

Auxin transport assays were conducted on light grown intact seedlings as previously described [100], with few modifications. *A.thaliana* 5-days-old seedlings, vertically grown in Petri dishes, were used for both basipetal and acropetal analyses. Seedlings from the growth plate were transferred to the assay plate, containing agar at 0.8% (w/v). Radiolabelled ^3^H-IAA (Amersham Biosciences; specific activity of 962 GBq mmol^−1^) was added to 1.25% (w/v) agar solution to reach a final concentration of approximately 100 nM. The auxin source was used to prepare auxin-containing agar droplets. For root acropetal auxin transport, agar droplets were positioned just below the root-shoot junction, while for root basipetal auxin transport the auxin-containing agar was applied just below root apices and the agar carefully moved by means of a stereomicroscope until it overlapped the root tip by approximately 0.5 mm. For root acropetal auxin transport, plates were incubated for 18 h at room temperature in the dark, after being inverted in order to avoid the potential diffusion of ^3^H-IAA along the surface of the root. For root basipetal auxin transport, plates were placed at room temperature in the dark for 6 h. Radiolabelled auxin was quantified for acropetal auxin transport analysis by harvesting the apical 5 mm of root tip. For basipetal auxin transport analysis, 2 mm of the root closest to the site of auxin application was excised and discarded, and the subsequent 5-mm segment back from the root tip was recovered. Each section was placed in scintillation fluid (Perkin-Elmer) and then analysed in a Beckman scintillation counter (Beckman Instruments). The amount of auxin transported (fmol) into each segment of wild-type and *ataucsia*-1 mutant was compared in Student's t-test.

### Yeast Two-hybrid

Yeast two-hybrid screens were performed on a 7-day-old *A. thaliana* seedlings cDNA library; the complete *AtAucsia*-1 sequence was used as bait. The screens were carried out by Hybrigenics (Paris, France) using its standard procedures. Relevance of each identified interaction was noted on an A to E predicted biological score scale as follow. “A,” “B,” and “C” show “very high,” “high,” and “good” confidence, respectively, in the interaction. D indicates moderate confidence.

### Recombinant GST- and His-tagged Proteins

The full length *AtAucsia*-1 coding sequence was PCR amplified using as template cDNA obtained by reverse transcribing mRNA extracted from *A. thaliana* seedlings. The upstream primer was 5′–GGATCCATGGCGCCACCTCCAGGACTTTA–3′ (*Bam*HI site is underlined) and the downstream primer was 5′–CTCGAGTTAGTGATGAGCCTTTGCTTCGGC–3′ (*Xho*I site is underlined). The PCR product was double-digested with *BamH*I *and Xho*I and cloned into pGEX4T1 (Amersham Biosciences), resulting in the in-frame fusion with the glutathione S-transferase (GST) sequence. The recombinant vector was checked by sequencing.

The DNA sequence spanning from nucleotide +6430 to nucleotide +6984 the coding region of *At4g31570* (i.e AtAUCSIA-1IP) was PCR amplified with the upstream primer 5′-CATATG *CATCATCATCATCATCAG*ATGGAAATTTGTGGTTCTCTCTCCCAA-3′ (*Nde*I site is underlined, (His)_6_-tag is in italic) and with the downstream primer 5′-GGATCCTTATTAGGCACTTGCAGATTGAAGATCTTC-3′ (*Bam*HI site is underlined). The PCR product was double-digested with *Nde*I and *BamH*I, cloned into pET12b (Novagen), and checked by sequencing.

The DNA sequence spanning from nucleotide +1 to nucleotide +443 the coding region of *At1g05010* (i.e ACO2) was PCR amplified with the upstream primer 5′- CATATG *CATCATCATCATCATCAC*GAGAGTTTCCCGATCATCAAT-3′ (*Nde*I site is underlined, (His)_6_-tag is in italic) and with the downstream primer 5′- GGATCCTTATTAGTAAAACACCTTTTTTAAATAACC-3′ (*Bam*HI site is underlined). The PCR product was double-digested with *Nde*I and *BamH*I, cloned into pET12b (Novagen), and checked by sequencing.

The resulting recombinant vectors were mobilized into the host strain *E.coli*, BL21(DE3)pLysS and the expression of recombinant proteins was induced by 0.05 mM isopropyl-β-D-thiogalactosidase at 16°C for 16 hours. AtAUCSIA1-GST, (His)_6_- AtAUCSIA-1IP and (His)_6_-ACO2 were purified through glutathione-agarose immobilized column (Thermo Scientific) and through nickel-nitrilacetic acid (Ni-NTA) agarose magnetic beads (QIAGEN), respectively, according to the manufacturers' protocols.

### Pull-down Binding Assay

Approximately 50 µg of either (His)_6_- AtAUCSIA-1IP or (His)_6_- ACO2 fusion protein was bound to nickel-nitrilotriacetic acid (Ni-NTA) magnetic agarose beads (QIAGEN) and incubated with 50 µg of GST:AtAUCSIA-1 fusion protein in the interaction buffer (300 mM NaCl, 10 mM Tris-HCl, 20 mM Imidazole, pH 8) for 2 hours at room temperature on an end-over-end shaker. After three washes with the interaction buffer, the (His)_6_-tagged proteins and its interactor were eluted with the same buffer containing 400 mM imidazole. The eluted proteins were separated on a 12% SDS-PAGE and electroblotted to a PVDF membrane (Millipore). The membrane was probed with either anti-(His)_6_ (Sigma) or anti-GST antibody (Thermo Scientific) to reveal the presence of the two interacting partners. The antigen-antibody complexes were developed using a chemioluminescent system.

### Isolation of *Arabidopsis* Mesophyll Protoplasts and BiFC Assay

Mesophyll *A.thaliana* protoplasts were prepared according to Abel and Theologis [101]. Briefly, rosetta leaves were harvested from 3- to 4-week-old plants and incubated with the protoplasting solution (1% Cellulase “Onozuka” R-10, 0.25% Macerozyme R-10, 0.5 mM PMSF, 400 mM Mannitol, 8 mM CaCl_2_, 5 mM MES-KOH, 0.1% BSA, pH 5.6) for 4 h on a vertical shaker at room temperature. The protoplast solution was filtered through a 150 µm nylon mesh and sedimented by centrifugation at 60×g for 3 min. The pellet was washed in WIMK solution (500 mM Mannitol, 5 mM MES, pH 5.6) and resuspended in MaMg-buffer (400 mM Mannitol, 15 mM MgCl_2_, 5 mM MES, pH 5.6). The protoplast solution was kept at 4°C for at least 30 min prior transfection.

The BiFC assays in *A. thaliana* protoplasts were carried out as described by Walter and colleagues [102]. The coding sequence of *AtAucsia*-1 and the ORF portions of *At4g31570* were PCR amplified and cloned in both pUC-SPYNE and pUC-SPYCE vectors [102]. To address the reliability of BiFC data, a positive control was introduced in the experimental procedure and it was constituted by the combination of a pUC-SPYNE and a pUC-SPYCE both containing the bZIP63 protein, which is an *A.thaliana* transcription factor well known for the formation of homodimers and heterodimers via C-terminal leucine zipper domain.

Approximately 15 µg of plasmid DNA of each construct were added to the protoplasts suspension along with the PEG solution (40% PEG 4000, 300 mM CaCl_2_, 5 mM MES, pH 5.6) to a final PEG concentration of 20% and the suspension was carefully mixed by hand until homogeneous. Following incubation at room temperature for 3–5 min, the transfection mixture was carefully diluted with WIMK solution. Protoplasts were collected (4 min at 60×g) and washed once in WIMK solution. Transfected protoplasts were incubated in the dark at 22°C for 16 h and the fluorescence emission was checked through a confocal laser scanning microscope (Fluoview FV1000; Olympus).

### Statistical analysis

The mean values ± standard error (SE) are reported in the figures. Statistical analyses were conducted using a Student's *t* test.

## Supporting Information

Figure S1 **Pairwise alignment of** ***AtAucsia*** **-1 and** ***AtAucsia*** **-2 coding sequences.**
(TIF)

Figure S2 **1-NPA sensitivity of** ***ataucsia*** **-1 mutant (SALK_ 117986).** (A) 5 days old seedlings were treated with 0.5 and 0.1 µM 1-NPA for 5 days. Density of emerged lateral roots is shown. (B) 5 days old seedlings were cultivated in the presence of 3 and 30 µM 1-NPA. Primary root length was measured after 5 days. The values reported in the panels are means ± standard error (SE) (n = 3) 15–20 seedlings per replicate. Data reported were analyzed by Student's *t*-test. **P<0.01; ***P<0.001 versus wild-type seedlings.
(TIF)

Figure S3 **AtAucsia-1OX #2 root and floral phenotypes.** (A) Quantification of IAA treatment (0.03 and 0.1 µM) effect on primary root growth of 10 days old wild-type and AtAucsia-1OX #2 seedlings (B). Flower at stage 13 (anthesis) of wild-type and AtAucsia-1OX #2 overexpressing line. In wild type flower, anthers are positioned above the stigma and pollen grains are released (upper panel). In AtAucsia-1OX #2 flowers (lower panel), some developmental defects were visible in the stamens. Arrows highlight stamens with an abnormal orientation, stamen filament fusion and stamen filament of reduced length.
(TIF)

Figure S4 ***AtAucsia*** **-1 and** ***AtAucsia*** **-2 promoter sequences and Pro_AtAucsia-2_::GUS analysis.** (A) Promoter sequence of *AtAucsia*-1 gene. (B) Promoter sequence of *AtAucsia*-2 gene. (C) Histochemical analysis of GUS activity driven by the *AtAucsia*-2 promoter in *A. thaliana*. 1. GUS activity in embryo at mature cotyledon stage; 2. 2–3 days after germination seedling; 3. 5–7 days old seedling. Arrows indicate GUS activity in both lateral and primary root tips; 4. flower at stage 11 of development; 5. Ovule funiculi; 6. anther and mature pollen grains.
(TIF)

Figure S5 **AtAUCSIA-1 interacts** ***in vitro*** **with 1-aminocyclopropane-1-carboxylate oxidase (ACO2;** ***At1g62380*** **).** (A) ACO2 protein was identified by yeast two-hybrid assay. Six independent clones, corresponding to portions of ACO2 protein, interacted with the entire AtAUCSIA-1 protein (green box) defining a common overlapping domain of 148 aminoacids (red box). (B) The GST-AUCSIA-1 fusion protein interacts with (His)_6_-ACO2 in an *in vitro* binding assay. (His)_6_-ACO2 protein, either in denatured (den (His)_6_-ACO2) or native form, was bound to nickel-nitrilotriacetic acid magnetic beads and then incubated in the presence of purified GST-AUCSIA-1. The protein fractions bound to magnetic beads were eluted, separated by SDS-PAGE, and detected by Western blot using either anti-GST or anti-His antibodies. (His)_6_-ACO2 coated beads pulled down GST-AUCSIA-1. Beads alone were used as negative control to test non specific interactions. Purified (His)_6_-ACO2 and GST-AUCSIA-1 proteins were used as protein markers in SDS-PAGE.
(TIF)
