# Supplementary figures and images for: Arabidopsis thaliana AUCSIA-1 Regulates Auxin Biology and Physically Interacts with a Kinesin-Related Protein
Source: PLoS One. 2012 Jul 20;7(7):e41327. doi: 10.1371/journal.pone.0041327 (PMC3401106; doi:10.1371/journal.pone.0041327)

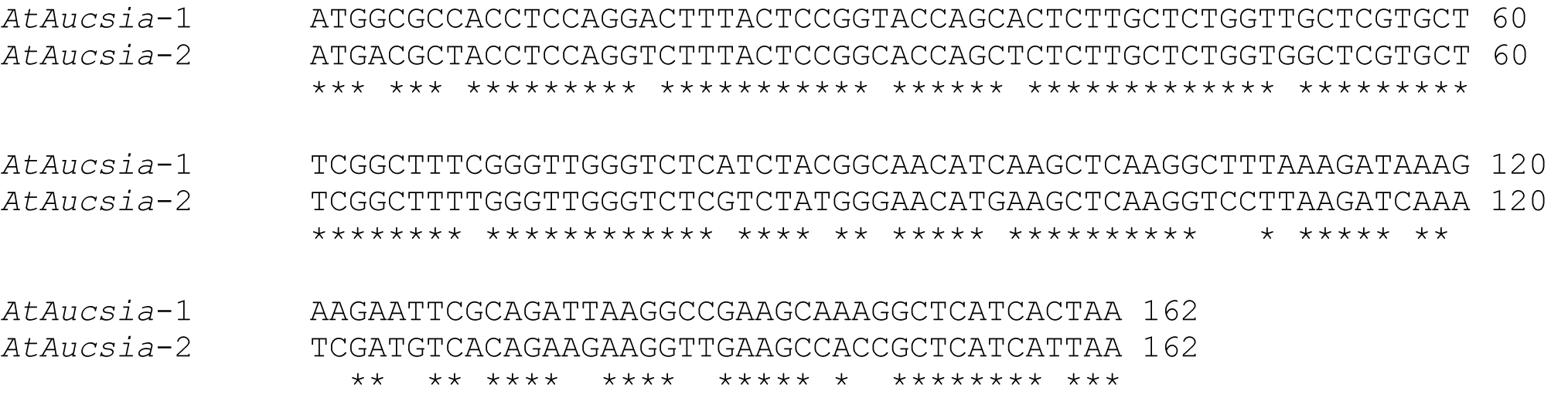

Supplement: Figure S1 — Pairwise alignment of AtAucsia -1 and AtAucsia -2 coding sequences. (TIF) [file pone.0041327.s001.tif]

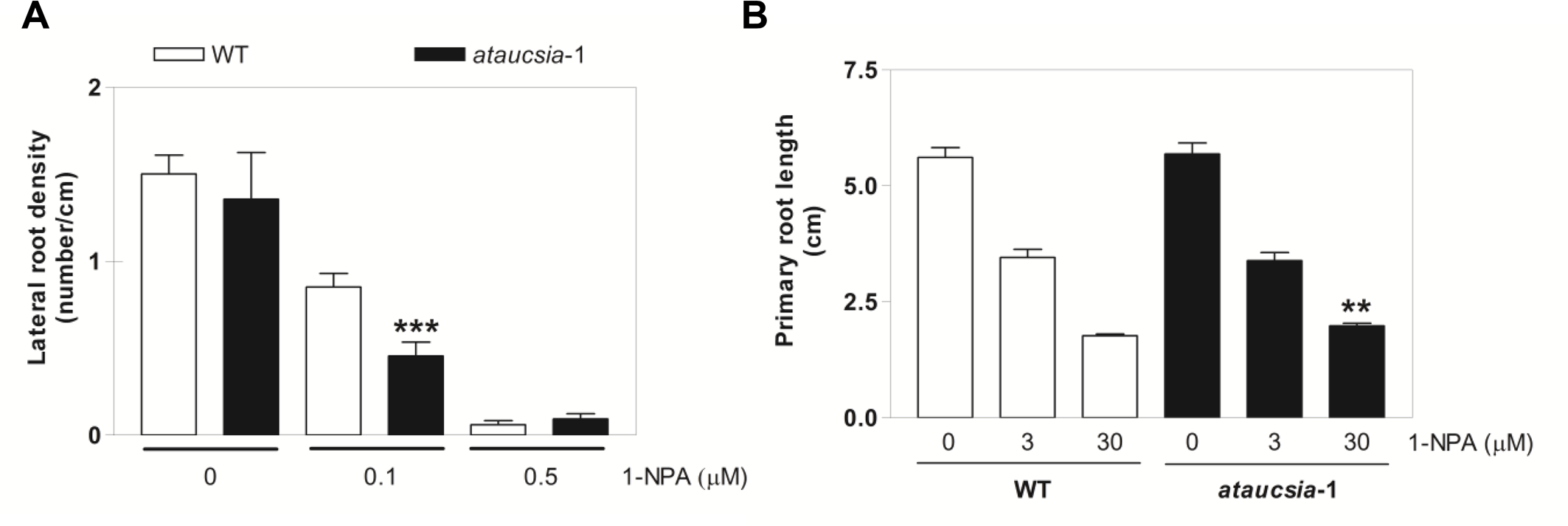

Supplement: Figure S2 — 1-NPA sensitivity of ataucsia -1 mutant (SALK_ 117986). (A) 5 days old seedlings were treated with 0.5 and 0.1 µM 1-NPA for 5 days. Density of emerged lateral roots is shown. (B) 5 days old seedlings were cultivated in the presence of 3 and 30 µM 1-NPA. Primary root length was measured after 5 days. The values reported in the panels are means ± standard error (SE) (n = 3) 15–20 seedlings per replicate. Data reported were analyzed by Student's t-test. **P<0.01; ***P<0.001 versus wild-type seedlings. (TIF) [file pone.0041327.s002.tif]

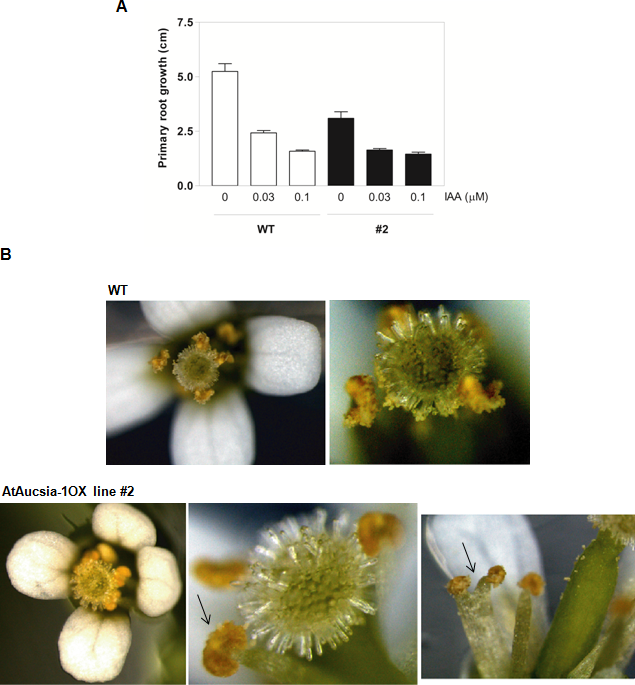

Supplement: Figure S3 — AtAucsia-1OX #2 root and floral phenotypes. (A) Quantification of IAA treatment (0.03 and 0.1 µM) effect on primary root growth of 10 days old wild-type and AtAucsia-1OX #2 seedlings (B). Flower at stage 13 (anthesis) of wild-type and AtAucsia-1OX #2 overexpressing line. In wild type flower, anthers are positioned above the stigma and pollen grains are released (upper panel). In AtAucsia-1OX #2 flowers (lower panel), some developmental defects were visible in the stamens. Arrows highlight stamens with an abnormal orientation, stamen filament fusion and stamen filament of reduced length. (TIF) [file pone.0041327.s003.tif]

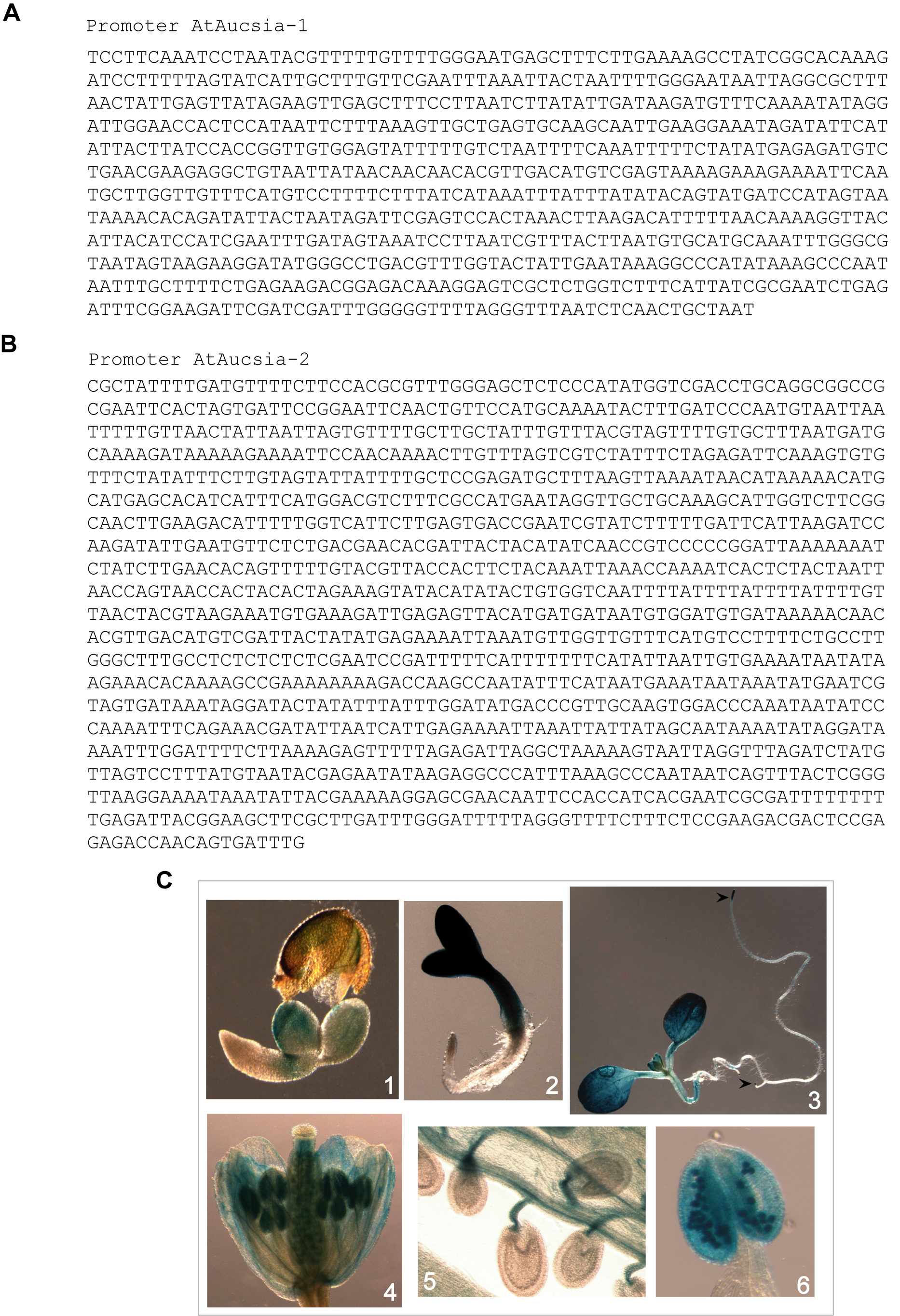

Supplement: Figure S4 — AtAucsia -1 and AtAucsia -2 promoter sequences and ProAtAucsia-2::GUS analysis. (A) Promoter sequence of AtAucsia-1 gene. (B) Promoter sequence of AtAucsia-2 gene. (C) Histochemical analysis of GUS activity driven by the AtAucsia-2 promoter in A. thaliana. 1. GUS activity in embryo at mature cotyledon stage; 2. 2–3 days after germination seedling; 3. 5–7 days old seedling. Arrows indicate GUS activity in both lateral and primary root tips; 4. flower at stage 11 of development; 5. Ovule funiculi; 6. anther and mature pollen grains. (TIF) [file pone.0041327.s004.tif]

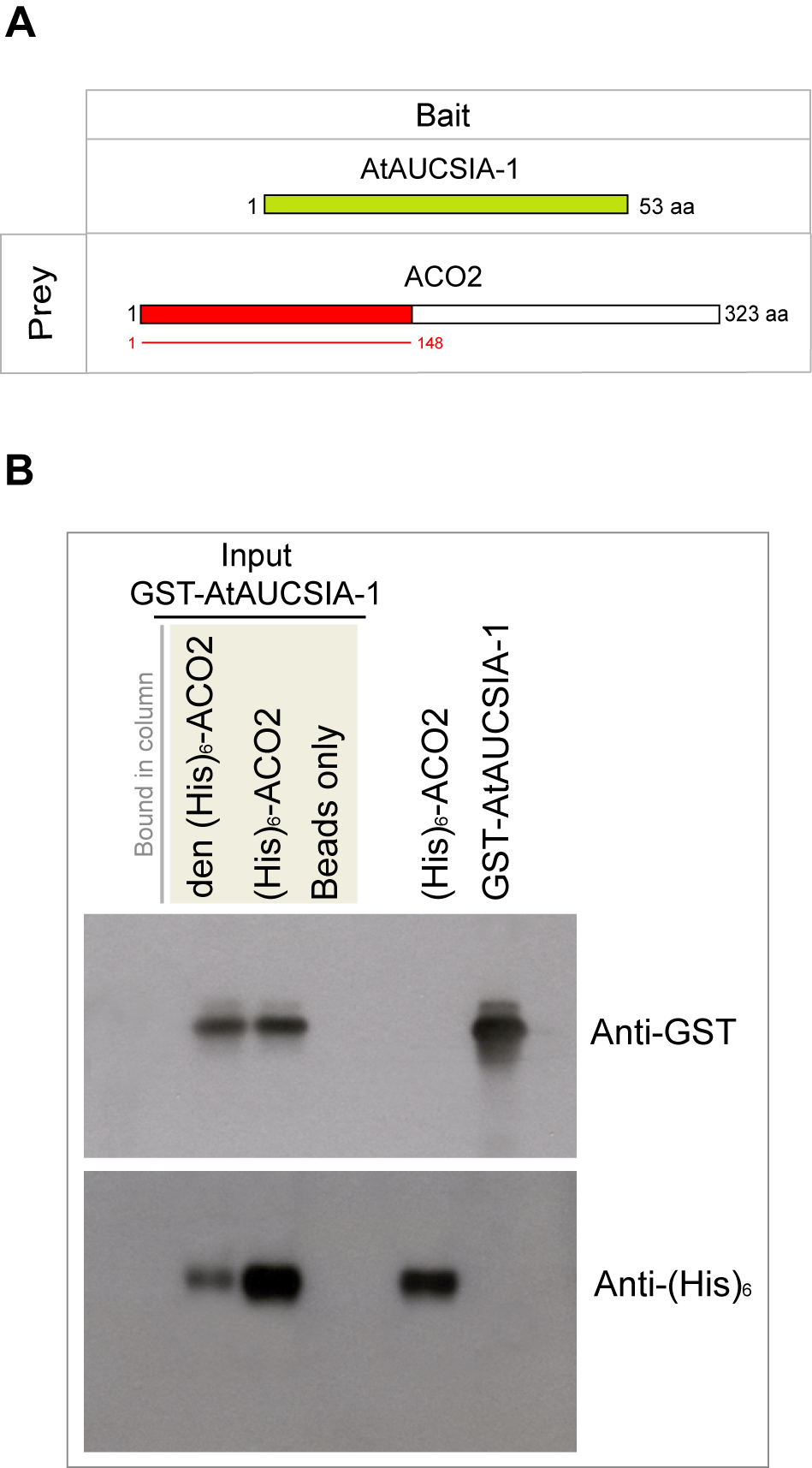

Supplement: Figure S5 — AtAUCSIA-1 interacts in vitro with 1-aminocyclopropane-1-carboxylate oxidase (ACO2; At1g62380 ). (A) ACO2 protein was identified by yeast two-hybrid assay. Six independent clones, corresponding to portions of ACO2 protein, interacted with the entire AtAUCSIA-1 protein (green box) defining a common overlapping domain of 148 aminoacids (red box). (B) The GST-AUCSIA-1 fusion protein interacts with (His)6-ACO2 in an in vitro binding assay. (His)6-ACO2 protein, either in denatured (den (His)6-ACO2) or native form, was bound to nickel-nitrilotriacetic acid magnetic beads and then incubated in the presence of purified GST-AUCSIA-1. The protein fractions bound to magnetic beads were eluted, separated by SDS-PAGE, and detected by Western blot using either anti-GST or anti-His antibodies. (His)6-ACO2 coated beads pulled down GST-AUCSIA-1. Beads alone were used as negative control to test non specific interactions. Purified (His)6-ACO2 and GST-AUCSIA-1 proteins were used as protein markers in SDS-PAGE. (TIF) [file pone.0041327.s005.tif]
